# Fifty years of oxacalix[3]arenes: A review

**DOI:** 10.3762/bjoc.8.22

**Published:** 2012-02-07

**Authors:** Kevin Cottet, Paula M Marcos, Peter J Cragg

**Affiliations:** 1 School of Pharmacy and Biomolecular Sciences, Huxley Building, University of Brighton, Brighton BN2 4GJ, UK; 2 UFR de Chimie, Université Joseph Fourier Grenoble 1, 301 rue de la Chimie, BP53 - 38041 Grenoble Cedex 9, France; 3 Centro de Ciências Moleculares e Materiais, FCUL, Edifício C8, 1749-016 Lisboa, Portugal and Faculdade de Farmácia da Universidade de Lisboa, Av. Prof. Gama Pinto, 1649-003 Lisboa, Portugal

**Keywords:** calixarenes, host–guest chemistry, macrocycles, oxacalixarenes

## Abstract

Hexahomotrioxacalix[3]arenes, commonly called oxacalix[3]arenes, were first reported in 1962. Since then, their chemistry has been expanded to include numerous derivatives and complexes. This review describes the syntheses of the parent compounds, their derivatives, and their complexation behaviour towards cations. Extraction data are presented, as are crystal structures of the macrocycles and their complexes with guest species. Applications in fields as diverse as ion selective electrode modifiers, fluorescence sensors, fullerene separations and biomimetic chemistry are described.

## Introduction

**Figure 1:**
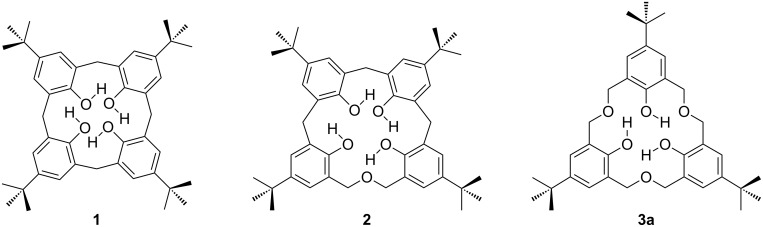
Calixarenes and expanded calixarenes: *p*-*tert*-Butylcalix[4]arene (**1**), *p*-*tert*-butyldihomooxacalix[4]arene (**2**), *p*-*tert*-butylhexahomotrioxacalix[3]arene (**3a**).

**Figure 2:**
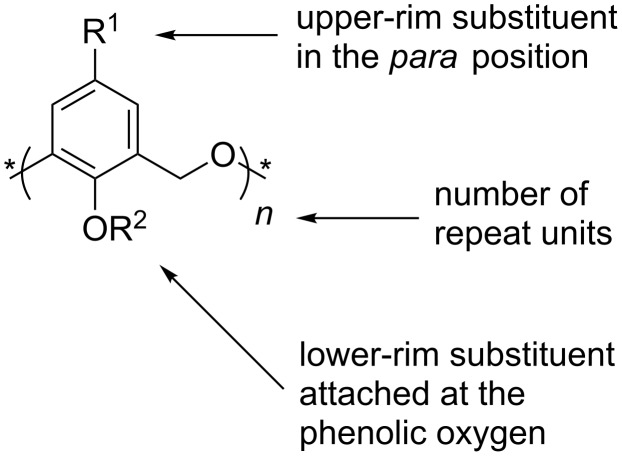
Conventional nomenclature for oxacalix[*n*]arenes.

Calixarenes, macrocycles which are widely used in supramolecular chemistry, are 2,6-metacyclophanes with a methylene bridge between their phenolic groups, as shown in Figure 1 [1–3]. In 1994, the term “homocalixarene” was coined by Brodesser and Vögtle to describe analogues of calixarenes with two or more methylene groups between the aromatic moieties [4]. When one or more CH_2_ bridges are replaced by CH_2_OCH_2_ groups the macrocycles are known as homooxacalixarenes, or simply oxacalixarenes. The presence of the heteroatom is reflected in the name of the compound, for example, *p*-*tert*-butylcalix[4]arene (**1**) with a CH_2_OCH_2_ group instead of a CH_2_ bridge is *p*-*tert*-butyldihomooxacalix[4]arene (**2**) [5]. “Dihomo” implies two additional atoms in the bridge and “oxa” that one of them is oxygen. The remainder of the calixarene nomenclature denotes any substituents attached to the phenolic oxygens, known as the “lower rim”, and substituents found in the *para*-position of the phenols, also known as the “upper rim” (Figure 2). For the purposes of this review the term “oxacalix[*n*]arene” will be used as a generalization for this class of compounds.

Although some aspects of homooxacalixarene chemistry have been reviewed [4,6–8], notably by Shokova and Kovalev in 2004 [9–10], it is timely for the 50^th^ anniversary of Hultzsch’s discovery of *p*-*tert*-butylhexahomotrioxacalix[3]arene (**3a**) [11] to reflect on the history of these compounds and assess recent advances in the field. Many other expanded calix[*n*]arenes are now known, including the methyl ethers of dihomooxa-, tetrahomodioxa-, hexahomotrioxa- and octahomotetraoxacalix[4]arenes, which have been described in detail by Masci [12]. Despite these advances, the oxacalix[3]arenes have remained the main focus of attention for researchers and are the subject of this review.

## Review

### 1 Synthesis of parent oxacalix[3]arenes

#### 1.1 Thermal dehydration

**Scheme 1:**
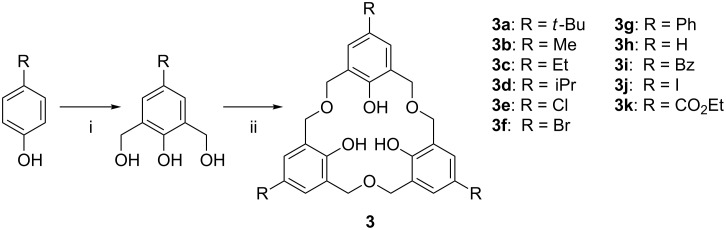
Synthesis of oxacalix[3]arenes: (i) Formaldehyde (37% aq), NaOH (aq), 1,4-dioxane; glacial acetic acid, acetone; (ii) refluxing *o*-xylene [14] or Na_2_SO_4_, MsOH, in refluxing DME [15].

The first oxacalix[*n*]arenes to be reported were the hexahomotrioxacalix[3]arenes, and these remain the most-studied members of the class. *p*-*tert*-Butylhexahomotrioxacalix[3]arene (**3a**), initially reported by Hultzsch in 1962, was isolated in less than 1% yield by heating 2,6-bis(hydroxymethyl)-4-*tert*-butylphenol [11]. Elemental analysis gave an empirical formula of C_12_H_16_O_2_ and molecular weight determinations gave values corresponding to a trimer. Despite interest in novel phenol–formaldehyde polymers and macrocycles and characterization of **3a** in 1979 [13], it took a further 20 years for a reproducible synthesis to be published. In 1983, Gutsche reported that the thermally induced dehydration of 2,6-bis(hydroxymethyl)phenols in xylene under reflux gave rise to the formation of homooxacalixarenes, some of them in reasonable yields, as shown in Scheme 1 [14].

**Figure 3:**
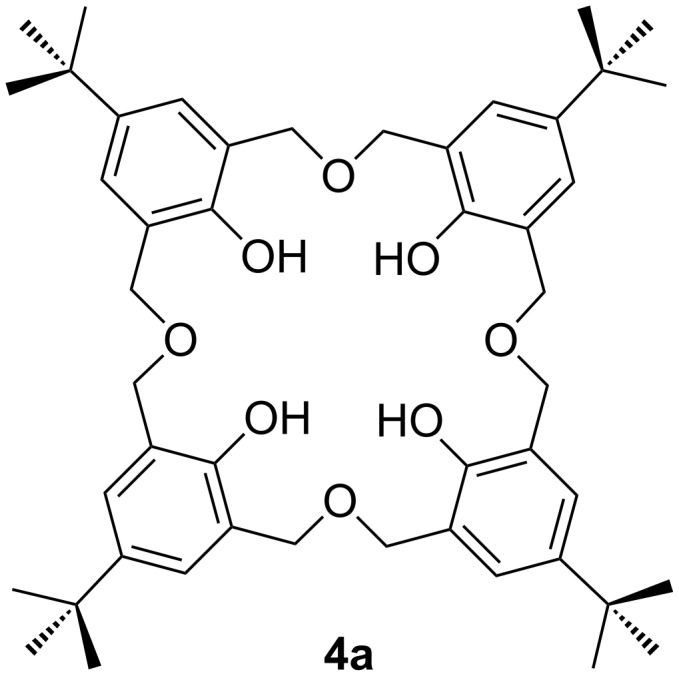
*p*-*tert*-Butyloctahomotetraoxacalix[4]arene (**4a**) [16].

Although not discussed by Gutsche, both cyclotrimers and tetramers are usually formed by this method and, in 1991, Vicens and Zerr performed a thermal dehydration of 2,6-bis(hydroxymethyl)-4-*tert*-butylphenol in xylene under reflux allowing them to isolate *p*-*tert*-butyloctahomotetraoxacalix[4]arene (**4a**), illustrated in Figure 3, along with **3a** [16].

**Figure 4:**
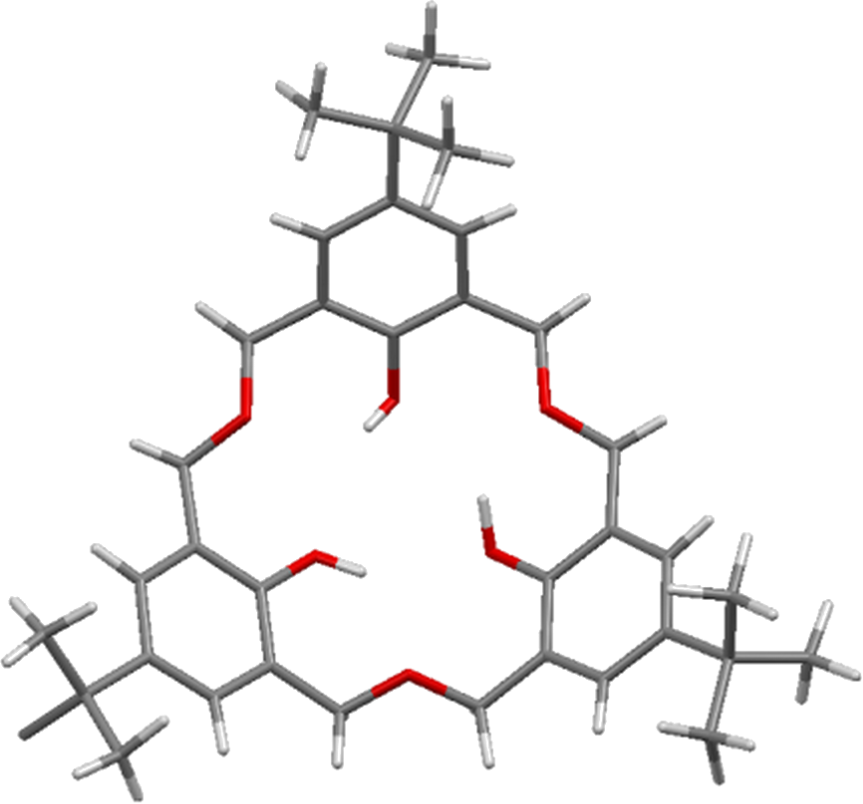
X-ray crystal structure of **3a** showing phenolic hydrogen bonding (IUCr ID AS0508) [17].

To finally prove that the main product from thermal dehydration was indeed a trimer, Vicens reported the X-ray crystal structure of **3a** in 1992 (Figure 4) demonstrating it to be exclusively in the bowl-shaped *cone* conformation [17].

In 1994, Hampton et al. used an alternative acid-catalyzed procedure to prepare **3a** and developed a method that improved its purity through the formation of the Na^+^ salt and its subsequent neutralization with acid [15]. The process separated **3a** from the cyclic tetramer; the former precipitates as the sodium salt in dry methanol due to complementarity between the arrangement of phenolic groups and the preferred coordination environment of Na^+^. Removal of the *tert*-butyl groups through a conventional AlCl_3_ driven retro-Friedel–Crafts de-*tert*-butylation reaction, as seen in other calixarenes, is unsuccessful in the case of oxacalixarenes, therefore different *para*-substituents must be introduced through the starting phenol in order to obtain derivatives with different groups at the upper rim. A number of other *para*-substituted bis(hydroxymethyl)phenols were therefore also cyclized in the presence of methanesulfonic acid (MsOH) or *para*-toluenesulfonic acid (TsOH) and Na_2_SO_4_. The corresponding oxacalixarenes were isolated in varying yields: *t*-Bu (**3a**) 32%; Me (**3b**) 21%; Et (**3c**) 21%; iPr (**3d**) 30%; Cl (**3e**)12% [15].

Although conditions were not necessarily optimal, the principles of oxacalix[3]arene syntheses had been established. Monomers react to give the cyclic trimer, predominantly, when heated under reflux in high-boiling-point organic solvents along with an organic acid. Water formed in the dehydration process must be removed through reaction with anhydrous drying agents or be collected in a Dean–Stark trap. In Gutsche’s report, and presumably in the work of Hultzsch too, the bis(hydroxymethyl)phenol monomer was isolated as the sodium salt and neutralized with acetic acid. Upon removal of solvent, traces of the acid presumably remained and were taken through to the cyclization step. Cragg noted that acid had to be present for the cyclization to occur, as carefully purified monomers formed calix[4]arenes or dihomooxacalix[4]arenes rather than oxacalix[3]arenes when subjected to standard synthetic methods [18]. To test this theory, the synthesis of **3a** was attempted in *o*-xylene under reflux by using either the freshly prepared crude monomer or the recrystallized monomer. The formation of **3a** was observed in the reaction of the unpurified monomer, but not under acid-free conditions. Moreover, in separate experiments MsOH, TsOH or glacial acetic acid (AcOH) were added to reactions involving the recrystallized monomer. MsOH or TsOH, having complementary threefold symmetry with the lower rim of oxacalix[3]arenes, were expected to increase the yields, but AcOH appeared to be just as effective. Notably, the addition of TsOH gave the oxacalix[3]arene as the sole product.

#### 1.2 Other synthetic methods

**Scheme 2:**
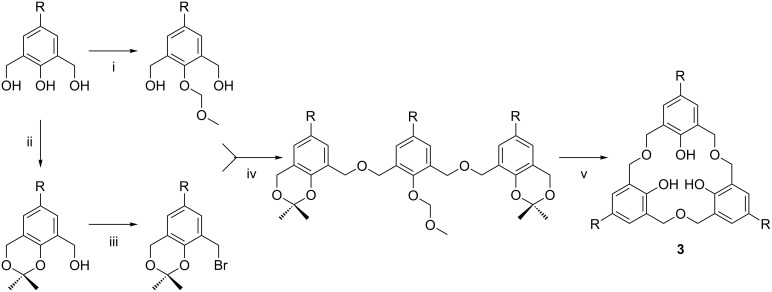
Stepwise synthesis of asymmetric oxacalix[3]arenes: (i) MOMCl, Adogen^®^464; (ii) 2,2-dimethoxypropane, *p*-TsOH; (iii) CBr_4_, PPh_3_, CH_2_Cl_2_; (iv) NaH, DMF; (v) HClO_4_ (aq), wet CHCl_3_ [19].

Since the initial reports of oxacalix[3]arene syntheses, several procedures have been developed to improve both the reaction conditions and the range of derivatives that can be prepared. The initial strategy to make oxacalix[3]arenes was a single step condensation, which can only lead to *C*_3_-symmetric compounds bearing the same *para*-substituted phenol; however, in host–guest chemistry an asymmetric macrocycle can provide a site for enantioselective molecular recognition. In the case of *p*-*tert*-butylcalix[*n*]arenes the *tert*-butyl substituent can be removed, as mentioned previously, through a retro-Friedel–Crafts acylation, and replaced by other groups, but the dibenzyl ether bridge in the oxacalixarenes is too fragile for this to be successful. In 1998, Fuji proposed a stepwise synthesis of asymmetric oxacalix[3]arenes based on linear precursors protected with a combination of isopropylidene and methoxymethyl groups [19]. As shown in Scheme 2, the phenolic position of a monomeric precursor is protected with methyl chloromethyl ether (MOMCl). A different monomer is then protected with 2,2-dimethoxypropane, in the presence of TsOH. This links one methylol group to the phenol, leaving the second open to bromination with CBr_4_ and PPh_3_. The linear trimer is formed between one methoxymethyl protected monomer and two benzyl bromide derivatives in DMF with NaH as the base. Intramolecular cyclization was achieved in 4 h at room temperature with 60% HClO_4_ in CHCl_3_ under high-dilution conditions. Pretreatment of the solvent with water was found to be necessary to remove the ethanol stabilizer and to aid deprotection. Yields were up to 50%, and, interestingly, there was no template effect from any alkali metals. An analogous strategy was developed by Georghiou in 2001 to prepare asymmetric oxacalix[3]naphthalene derivatives [20], and this is discussed in greater detail below.

**Figure 5:**
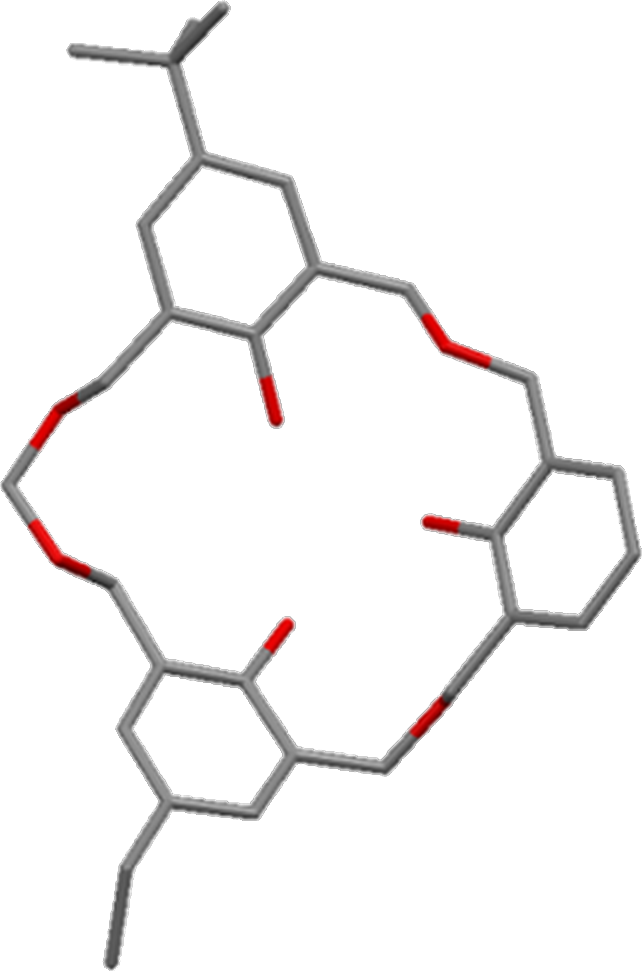
X-ray crystal structure of heptahomotetraoxacalix[3]arene **5** (CCDC ID 166088) [21].

In a later communication, Fuji reported the crystal structure of an unusual byproduct of the reaction, a heptahomotetraoxacalix[3]arene **5** with *t*-Bu, Et and H upper-rim substituents (Figure 5) [21].

**Scheme 3:**
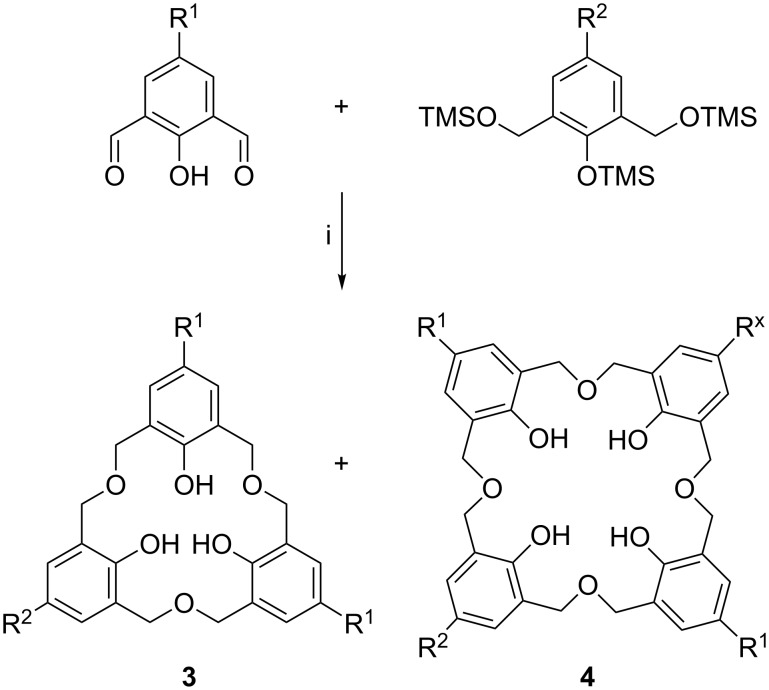
Oxacalix[3]arene synthesis by reductive coupling: (i) Me_3_SiOTf, Et_3_SiH, CH_2_Cl_2_; R^1^, R^2^ = I, Br, benzyl, *n*-octyl (x = 1 or 2) [22].

In 2001, Komatsu proposed a different way to access compounds in which two, or all three, units are identical [22]. The method was based on the reductive coupling of silylated derivatives of 2,6-hydroxymethylphenols, in which R is *t*-Bu, Me, benzyl (Bz), phenyl (Ph), or a halide, as shown in Scheme 3. The reaction takes place under conditions of high dilution at −78 °C to favour intramolecular cyclization over polymerization. Coupling reactions are successful, whether the groups in the *para*-position are the same or different, and this method also gives access to oxacalix[4]arenes in modest yields up to 42% for the *p*-*tert*-butyl derivative.

#### 1.3 Oxacalix[3]naphthalenes

**Scheme 4:**
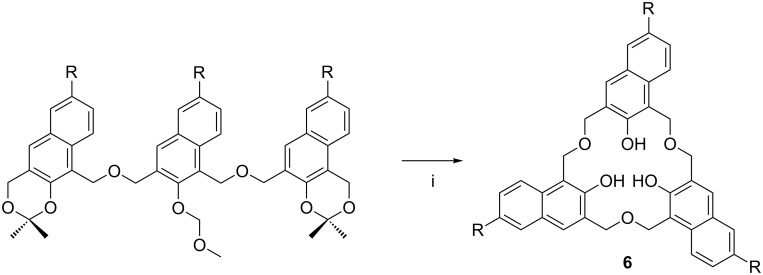
Oxacalix[3]naphthalene: (i) HClO_4_ (aq), wet CHCl_3_ (R = *tert*-butyl, **6a**, H, **6b**) [20].

The oxacalix[3]naphthalenes, e.g., **6a** and **6b** reported by Georghiou, have extended aromatic groups with H or *t*-Bu groups in the 6-position and can be considered as close relatives of the oxacalix[3]arenes [20]. The synthesis, shown in Scheme 4, is analogous to Fuji’s method for oxacalix[3]arenes [19]. As noted below, this extended aromatic surface is oriented perfectly for C_60_ inclusion [23].

### 2 Conformational properties

**Figure 6:**
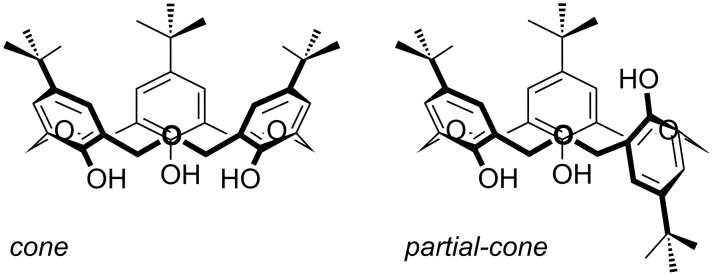
Conformers of **3a**.

Oxacalix[3]arenes have received significant attention as receptors, mainly due to their structural features: A cavity formed by a 18-membered ring, only two basic conformations (*cone* and *partial-cone*), and a *C*_3_-symmetry [24]. This last feature can provide a suitable binding site for species that require trigonal-planar, tetrahedral or octahedral coordination environments. The flexibility of the macrocycles can allow them to establish ideal bond distances and angles to bind such species. In common with other calix[*n*]arenes, oxacalix[3]arenes containing free OH groups are conformationally mobile, leading to *cone* and *partial-cone* conformers (Figure 6). Without lower-rim substituents there is free rotation of each phenolic unit through the macrocyclic annulus; however, the presence of a hydrogen-bond motif in the *cone* conformer makes it the more stable form.

In 1985, Gutsche investigated the conformational flexibility of parent calix[*n*]arenes (*n* = 4–8) and oxacalixarenes by temperature-dependent ^1^H NMR [5]. The *through-the-annulus* rotation barrier for oxacalix[3]arenes was calculated to be much lower than that for other calixarenes, either in non-coordinating or in polar solvents, such as CDCl_3_ or pyridine, respectively. The ^1^H NMR spectrum of **3a** in CDCl_3_/CS_2_ only showed a singlet for the CH_2_ resonance, even at −90 °C, and the ∆*G*^≠^ barrier for conformational inversion in CDCl_3_ was <38 kJ mol^−1^, in contrast with 66 kJ mol^−1^ for the calix[4]arene analogue. To freeze the oxacalix[3]arene conformer, *through-the-annulus* rotation must be prevented. This can be achieved by the introduction of sufficiently large groups on the lower rim of the macrocycle. Upper-rim inversion is less likely to occur when, as in the case of **3a**, it is hindered by the *tert*-butyl group.

### 3 Oxacalix[3]arene derivatives

#### 3.1 Lower-rim derivatives

**Scheme 5:**
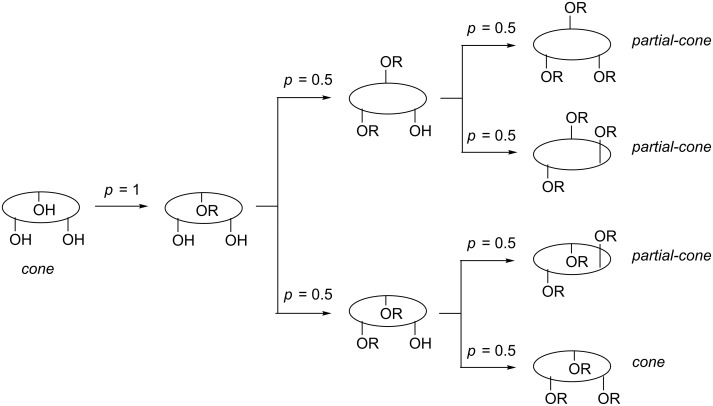
Origin of the 25:75 *cone*:*partial*-*cone* statistical distribution of *O*-substituted oxacalix[3]arenes (*p* = probability) [25–26].

Oxacalix[3]arene derivatization at the lower rim has been achieved through alkylation reactions with simple alkyl halides or with functionalized alkylating agents. Lower-rim derivatization is relatively straightforward, but conformational control is harder to achieve. The main drawback of lower-rim substitution is that statistically only 25% of the product is formed in the *cone* conformation, as shown in Scheme 5 [25–26].

**Scheme 6:**
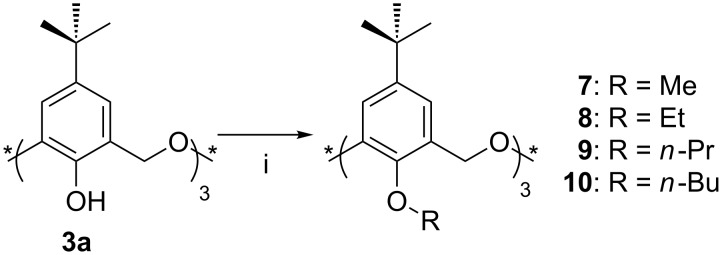
Synthesis of alkyl ethers **7**–**10**: (i) Alkyl halide, NaH, DMF [24].

**3.1.1 Alkyl ethers:** Classical *O*-alkylation of oxacalix[3]arenes was first achieved by Shinkai et al. in 1993 [24]. Treatment of **3a** with the corresponding alkyl halides in DMF in the presence of NaH afforded Me (**7**), Et (**8**), *n*-Pr (**9**) and *n*-Bu (**10**) derivatives (Scheme 6). Under these conditions, **8** was obtained in the *partial-cone* conformation only. When the reaction was performed in the presence of *t*-BuOK a 1:4 mixture of *cone* and *partial-cone* was obtained and even with Cs_2_CO_3_ the *cone* conformer could be detected. It seems that K^+^ and Cs^+^ favourably interact with the three phenolic oxygen atoms placed on the same side, whereas Na^+^ preferentially interacts with them across the ring.

**Scheme 7:**
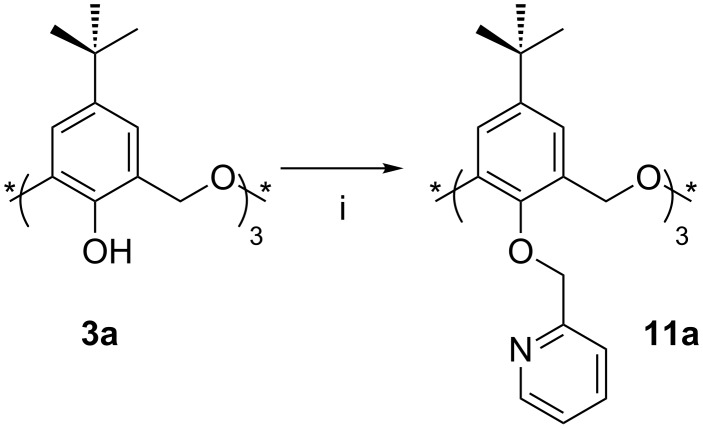
Synthesis of a pyridyl derivative **11a**: (i) Picolyl chloride hydrochloride, NaH, DMF [26–27].

Introduction of heteroatoms, such as nitrogen, into the oxacalix[3]arene lower rims can also be achieved by *O*-alkylation. Pyridine is known to be a good ligand towards metals and is widely employed in transition-metal coordination chemistry; therefore, in an attempt to incorporate these binding sites into oxacalix[3]arenes, Yamato [27] and Cragg [26] independently reacted **3a** with 2-(chloromethyl)pyridine, as shown in Scheme 7. The presence of Cs_2_CO_3_ leads to the formation of the *partial-cone* conformer, whereas K_2_CO_3_ and NaH increase the yield of the *cone* conformer of **11a** to about 25%. ^1^H NMR analysis of the *cone* conformer indicates that the nitrogen atoms point away from the macrocyclic cavity [27].

When 4-(chloromethyl)pyridine was used instead, NaH was ineffectual as a deprotonating agent. Na_2_CO_3_ yielded the disubstituted product only, K_2_CO_3_ gave both *cone* (8%) and *partial-cone* (68%) conformers, and the only isolated product with Cs_2_CO_3_ was the *partial-cone* conformer (75%) [28].

**Figure 7:**
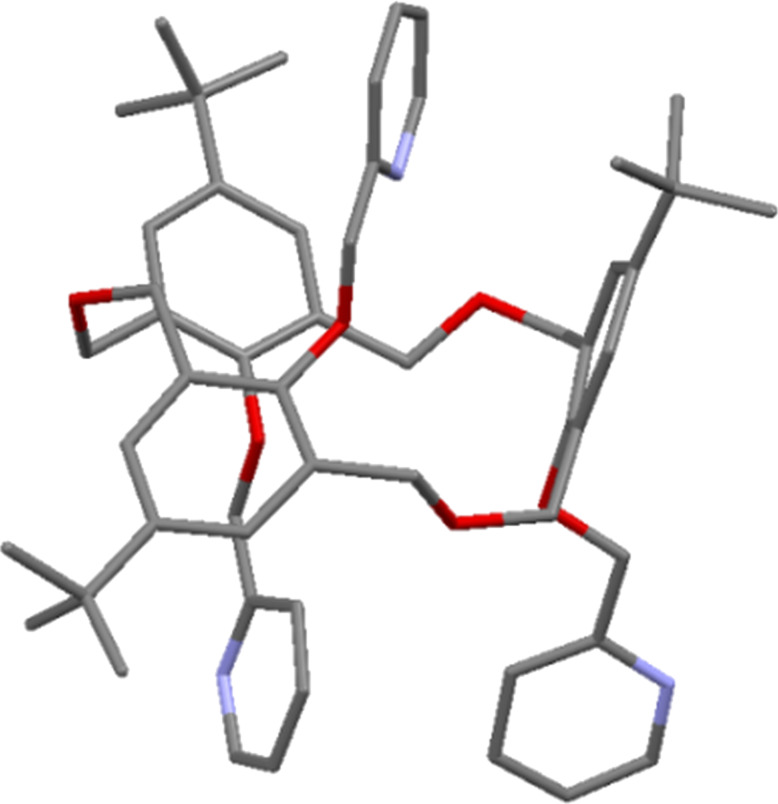
X-ray crystal structure of *partial-cone* **11a** (CCDC ID 150580) [26].

The X-ray structure of the *partial-cone* conformer (Figure 7), reported by Cragg, shows one pyridyl group to be included within the macrocyclic cavity and the remaining two with their nitrogen atoms pointing away from it [26].

**3.1.2 Functionalized alkyl ethers:** Functionalized alkyl halides of the type XCH_2_Y, where X is a leaving group and Y is a functional group, have also been used to introduce a variety of groups into the lower rim of oxacalix[3]arenes. Thus, derivatives containing carbonyl groups (ester, acid, amide and ketone) and heteroatoms, such as nitrogen and phosphorous, have been obtained.

**Scheme 8:**
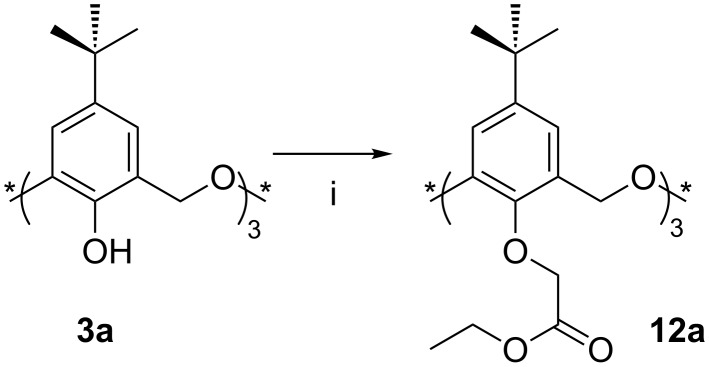
Lower-rim ethyl ester synthesis: (i) Ethyl bromoacetate, NaH, *t*-BuOK or alkali metal carbonate, THF or acetone [29].

In 1993, Shinkai et al. [29] reported the synthesis of the first ethyl ester derivative **12a**. In the belief that the alkali-metal template effect would lead preferentially to the *cone* conformer with NaH, the reaction of excess ethyl bromoacetate with **3a** was carried out in acetone under reflux (Scheme 8). The *partial-cone* conformer of **12a** was formed exclusively when weaker bases, K_2_CO_3_ or Cs_2_CO_3_, were used. NaH or *t*-BuOK in THF gave a mixture of products, but the yield of the *cone* conformer never exceeded 22%. An experiment with the oxacalix[3]naphthalene analogue was performed in 2003 by Georghiou [30], which also gave the *cone* conformer in 25% yield.

**Scheme 9:**
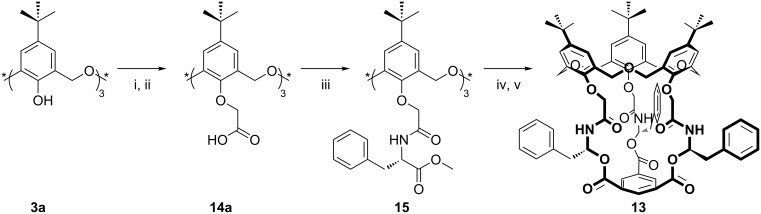
Forming chiral receptor **13**: (i) Ethyl bromoacetate, NaH, THF; (ii) NaOH, H_2_O/1,4-dioxane; (iii) *S*-PheOMe∙HCl, DCC, HOBt, NEt_3_, CH_2_Cl_2_; (iv) LiBH_4_, THF; (v) 1,3,5-benzenetricarbonyl chloride, pyridine, THF [31].

*Cone*-**12a** was used by Shinkai as the starting point from which to construct the chiral capped oxacalix[3]arene **13** as shown in Scheme 9 [31]. The parent compound was cleaved to form the tris(acid) **14a**, which then reacted with *S*-phenylalanine methyl ester. Deprotection of the methyl ester followed by reduction with LiBH_4_ gave the chiral amide **15**, which reacted with 1,3,5-benzenetricarbonyl chloride to form the capped species **13**. Compound **13** was shown to bind primary ammonium cations better than an uncapped ester analogue.

**Figure 8:**
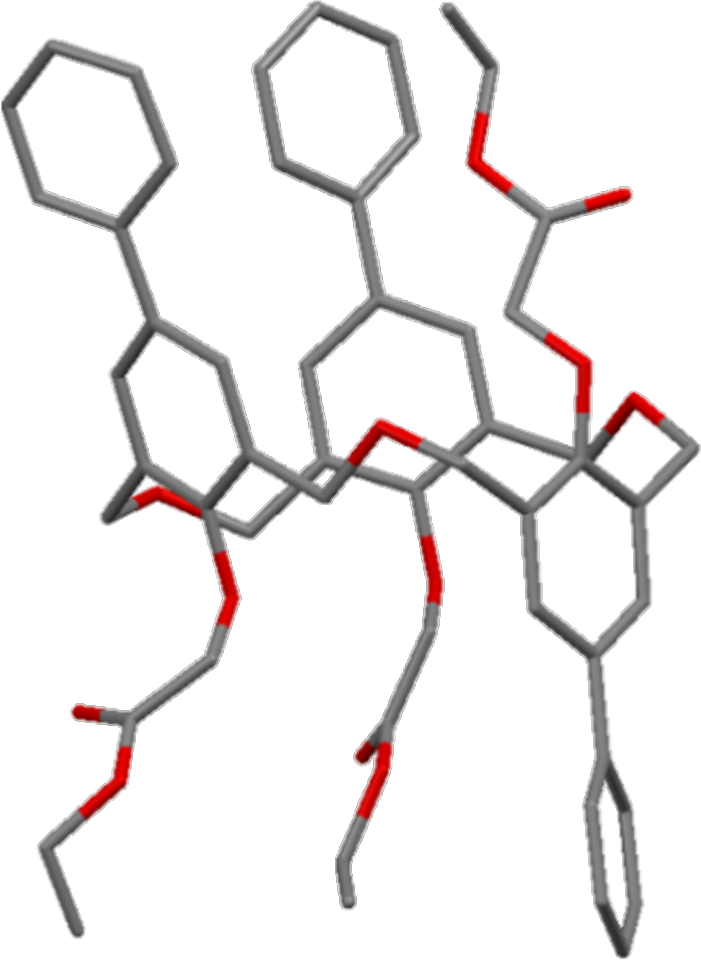
X-ray crystal structure of **16** (IUCr ID PA1110) [32].

In 1995, Vicens reported the crystal structure of a *partial-cone* triethyl ester derivative of 4-phenyloxacalix[3]arene illustrated as **16** in Figure 8 [32]. Few synthetic details were given; however, it was reported that cyclization of bis(2,5-methylol)-4-phenylphenol to give **3g** was followed by reaction with ethylbromoacetate, but no mention of the yield or isolation of a *cone* conformer was made.

**Scheme 10:**
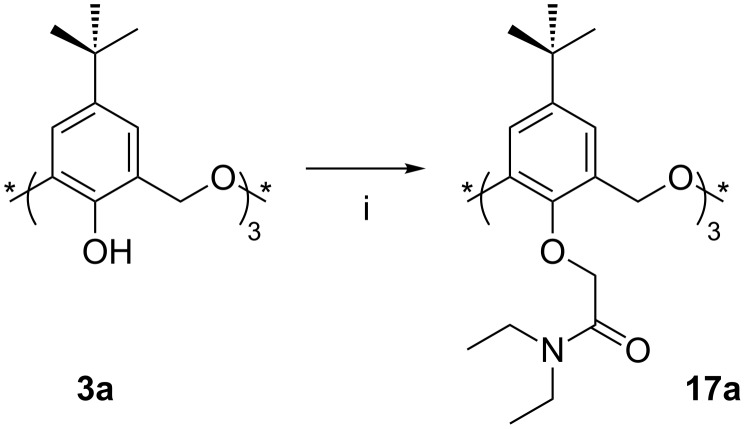
Lower rim *N*,*N*-diethylamide **17a**: (i) *N*,*N*-Diethylchloroacetamide, NaH, *t*-BuOK or alkali metal carbonate, THF or DMF or acetone [25–26,33].

The first amide derivative was reported by Shinkai in 1995 [25] through the reaction of **3a** with *N*,*N*-diethylchloroacetamide (Scheme 10). Heating under reflux in THF, with NaH as base, gave *cone* amide **17a** as the only isolated product in 23% yield. Using the same conditions, Cragg reported an improved yield of 44% through a slight modification of the previous procedure (recrystallization from MeCN instead of MeOH) [26], and Yamato later reported a 90% yield [33]. This is in stark contrast to the maximum yield of 25% for the esterification reaction discussed above and points to a subtle, yet essential, difference between the interaction modes of the oxacalixarene, cation and alkylating agent. Despite much speculation, the reason for this is not yet understood. As with the esterification reaction, use of K_2_CO_3_ or Cs_2_CO_3_ in place of NaH, and with acetone as the solvent, reverses the conformer preference with *partial-cone*-**17a** isolated in 45% yield with only a trace of the *cone* conformer. This suggests a template effect for both K^+^ and Cs^+^ that occurs whether an amide or ester is formed, and a function for Na^+^ beyond that of a mere template.

**Scheme 11:**
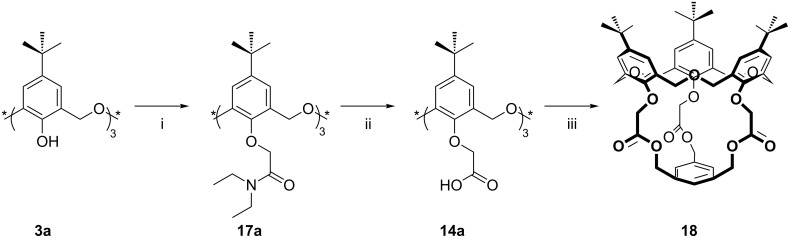
Capping the lower rim: (i) *N*,*N*-Diethylchloroacetamide, NaH, THF; (ii) NaOH, H_2_O/1,4-dioxane; (iii) 1,3,5-tris(bromomethyl)benzene, Na_2_CO_3_, DMF [33].

One consequence of this work is that the preferred route to *C*_3_ symmetric *cone* derivatives is through tris(amide) derivative **17a**, which can readily be cleaved by hydrolysis employing sodium hydroxide in 1,4-dioxane/water to give *cone*-**14a**. In 2001 Yamato used *cone*-**14a** to form a *C*_3_ symmetric hydrophobic receptor **18** in 13% yield through reaction with 1,3,5-tris(bromomethyl)benzene in the presence of Na_2_CO_3_ (Scheme 11) [33]. As the reaction failed to work when K_2_CO_3_ was used, the authors suggested that Na^+^ may play a templating role in addition to that of a deprotonating agent.

**Figure 9:**
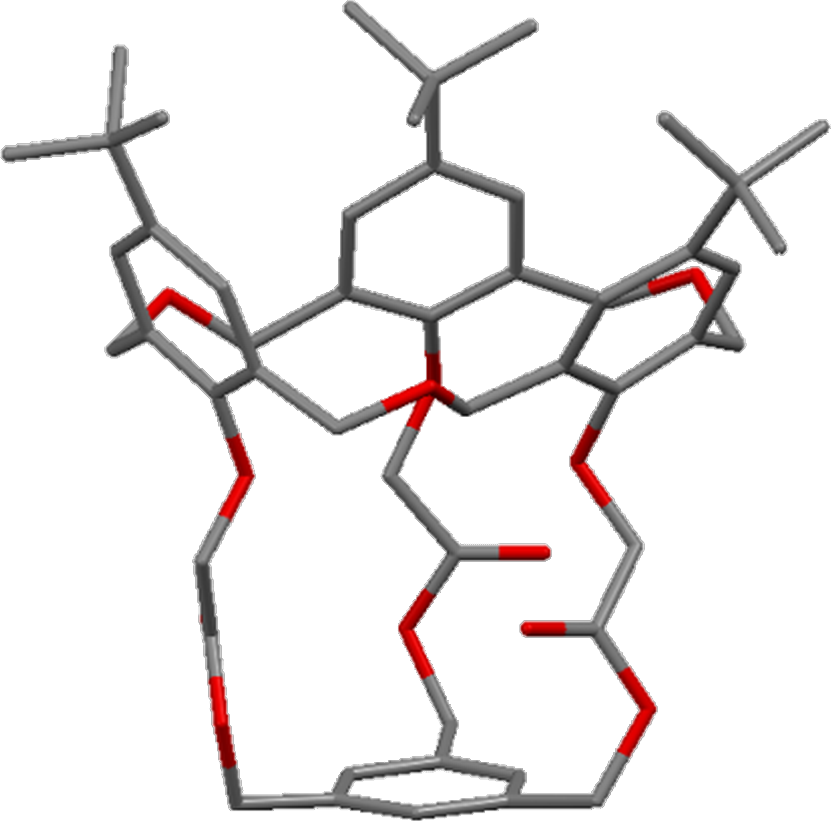
X-ray crystal structure of **18** (CCDC ID 142599) [33].

The X-ray crystal structure of the product (Figure 9) shows that the carbonyl oxygen atoms point away from the cavity to create a large hydrophobic cavity. Extraction studies indicated a slight, and statistically insignificant, preference for K^+^ over Cs^+^ and Ag^+^, with a much lower affinity for Na^+^. The highest affinity was reserved for *n*-BuNH_3_^+^.

An analogue of **18**, which showed little affinity for metal cations, was prepared with three 4-methylbenzyl substituents on the lower rim (**19**).

In 2001, Yamato reported an oxacalix[3]arene with pendant pyridines linked by amide bonds [34]. The intramolecular hydrogen bonds between neighbouring amide groups enforced a *flattened-cone* conformer for the macrocycle, which prevented binding to both metal cations and, to a large extent, alkyl ammonium cations. Extending the link between the macrocycle and aromatic termini did not disrupt the strong amide interactions, although binding was detected for Ag^+^, as the triflate, and for *n*-BuNH_3_^+^, as the chloride salt [35]. Further work on this class of derivatives showed some anion selectivity in the presence of *n*-BuNH_3_^+^ through intermolecular hydrogen bonding with amide hydrogens [36].

**Scheme 12:**
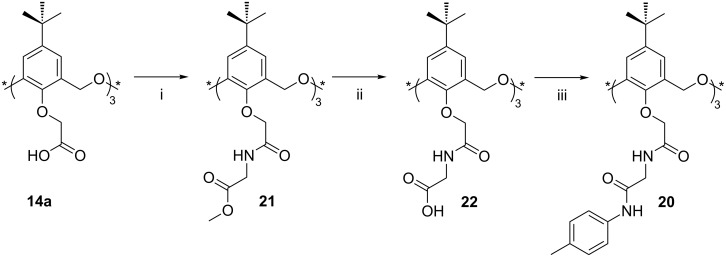
Extending the lower rim: (i) Glycine methyl ester, HOBt, dicyclohexycarbodiimide (DCC), CH_2_Cl_2_; (ii) NaOH, H_2_O/1,4-dioxane; (iii) *p*-toluidine, HOBt, DCC, CH_2_Cl_2_ [37].

In 2006, the same group used a similar route in order to synthesize the extended, uncapped derivative **20** incorporating three (phenylcarbamoyl)methylcarbamate substituents (Scheme 12), to mimic the binding sites in a protein, complete with hydrophobic region [37]. These amides were designed to act as heteroditopic receptors, capable of binding anions and cations separately and simultaneously in a cooperative way, and were shown to bind *n*-BuNH_3_^+^ halide salts in this manner.

**Scheme 13:**
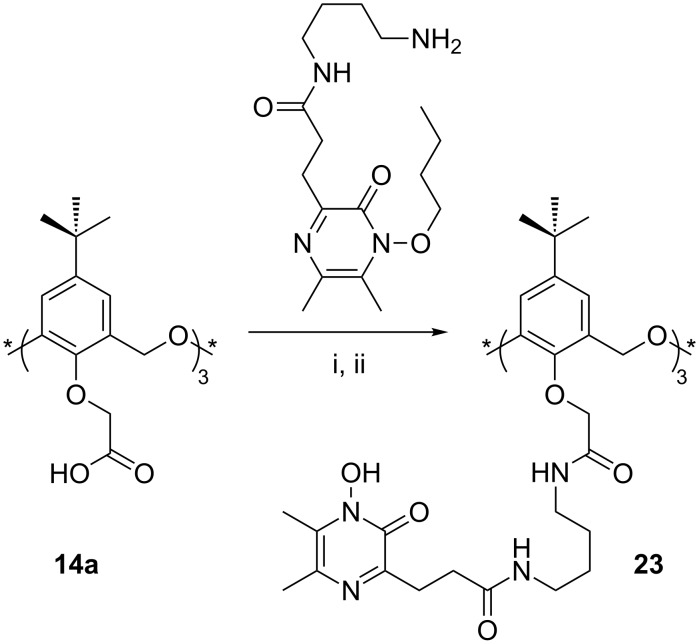
Synthesis of *N*-hydroxypyrazinone derivative **23**: (i) 1-[3-(Dimethylamino)propyl]-3-ethylcarbodiimide hydrochloride, HOBt, Et_3_N; (ii) H_2_, 10% Pd-C, MeOH [38].

*N*-Hydroxypyrazinones are known to function as bidentate ligands for metals such as iron or gallium that require an octahedral geometry. Katoh coupled *N*-hydroxypyrazinone substituents to *cone*-**14a** in order to prepare **23** (Scheme 13). Binding Ga^3+^ with remote lower-rim groups induced the cooperative binding of alkyl ammonium cations by the macrocycle [38].

**Scheme 14:**
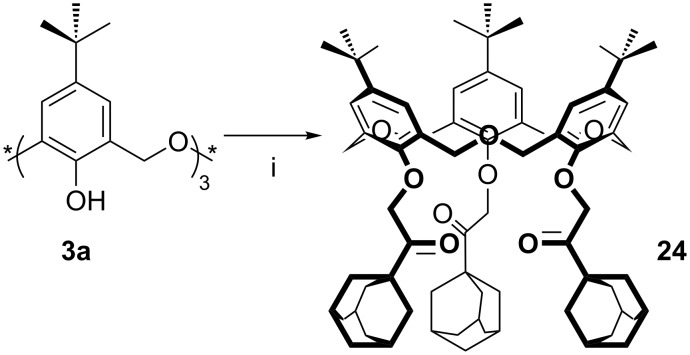
Synthesis of **24**: (i) 1-Adamantyl bromomethyl ketone, NaH, THF [39].

Recently, Marcos reported the synthesis of an oxacalix[3]arene ketone derivative (Scheme 14) [39]. Treatment of **3a** with 1-adamantyl bromomethyl ketone and NaH in THF under reflux afforded adamantyl ketone **24** in the *cone* conformation only.

**Scheme 15:**
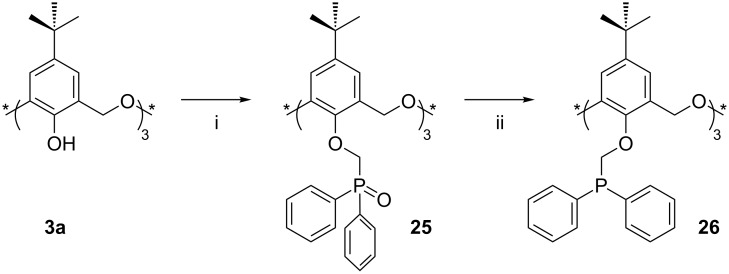
Synthesis of **25** and **26**: (i) (Diphenylphosphino)methyl tosylate, NaH, toluene; (ii) phenylsilane, toluene [40].

**3.1.3 Phosphorus derivatives:** Complete phosphorylation of **3a** was reported by Matt in 1999 and was achieved through reaction with NaH and Ph_2_P(O)CH_2_OTs in toluene at 90 °C for three days (Scheme 15) [40]. The reaction resulted in the formation of a 4:1 mixture of the *cone* and *partial-cone* diphenylphosphine oxide derivatives **25**: The preference for the *cone* formation is highly atypical but may be due to the templating effect of Na^+^. Separation by column chromatography afforded the *cone* conformer in 72% yield although the *partial-cone* was never obtained in a pure form. Reduction by phenylsilane (PhSiH_3_) gave the corresponding *cone* and *partial-cone* phosphines **26** quantitatively.

**Figure 10:**
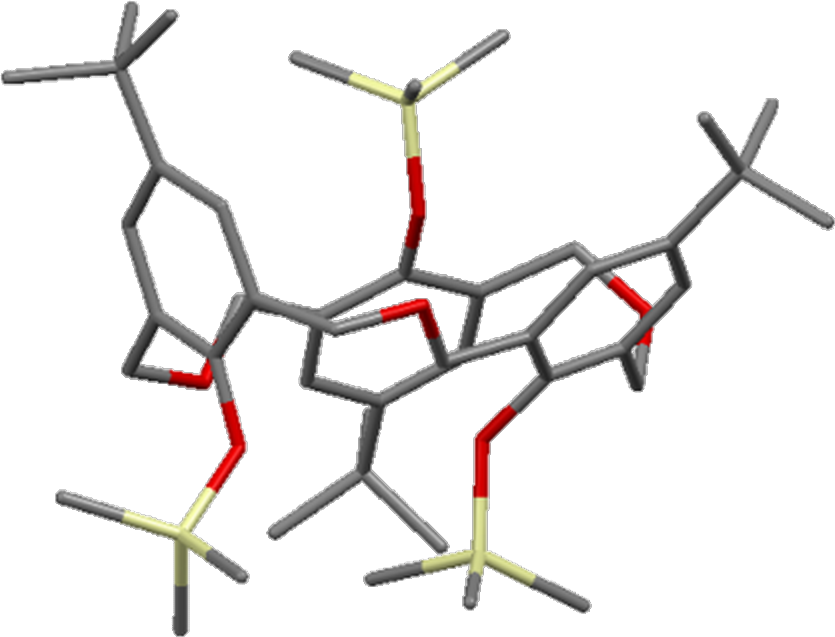
X-ray crystal structure of **27** in the *partial-cone* conformer (CCDC ID SUP 90399) [41].

**3.1.4 Silyl derivatives:** In 1996, Hampton investigated the selectivity of silylation on oxacalix[3]arenes to determine the influence of the group in the *para*-position, the nature of the silylating agent and the reaction conditions [41]. Unsurprisingly, the formation of the *partial-cone* was favoured for all oxacalix[3]arenes, with small upper-rim substituents having the highest *partial-cone*:*cone* ratio (e.g., 100:1 for the Cl derivative) when bis(trimethylsilyl)trifluoroacetamide was used as the silylating reagent. When 1-(trimethylsilyl)imidazole was used, the ratios were 30 to 45:1 and were independent of the group in the *para*-position. A silylated *p*-*tert*-butyloxacalix[3]arene **27** was characterized by X-ray crystallography to confirm that it was in the *partial-cone* conformation as shown in Figure 10. These derivatives could serve as reaction intermediates, due to the ease with which the silicon–oxygen bond can be cleaved in the presence of fluoride, although this chemistry has yet to be explored.

**Scheme 16:**
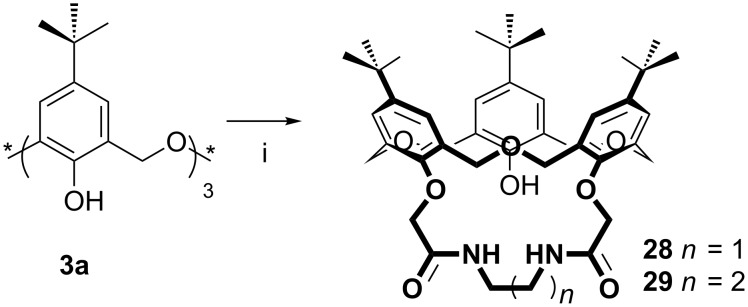
Synthesis of strapped oxacalix[3]arene derivatives **28** and **29**: (i) *N*,*N*’-Bis(chloroacetyl)-1,2-ethylenediamine or *N*,*N*’-bis(chloroacetyl)-1,3-propylenediamine, K_2_CO_3_, acetone [43].

**3.1.5 Intramolecularly bridged derivatives:** Linking two or more phenolic calixarene oxygen atoms together is a common method to improve selectivity and complex stability, and derivatives such as calixcrowns have been known for a considerable time [42]. Amido-di-*O*-bridged oxacalix[3]arenes were reported by Chen in 2005 through reaction of **3a** with *N*,*N’*-bis(chloroacetyl)-α,ω-alkylenediamines in refluxing acetone with K_2_CO_3_ as the base (Scheme 16) [43–44]. Those compounds linked by two (**28**) or three (**29**) methylene groups had a binding affinity for linear primary alkyl ammonium ions from *n*-BuNH_3_^+^ to *n*-HexNH_3_^+^.

#### 3.2 Upper-rim derivatives

Although the lower rim has many advantages as a binding site for guests, not least in the relative ease with which substituents can be attached, the upper rim can also function as a molecular recognition centre. The cavity created by the lipophilic phenolic units, particularly when held in place through allosteric effects of lower-rim substituents bound to metals, can accommodate a number of quaternary ammonium ions or buckminsterfullerene, C_60_. Consequently, the ability to vary the upper rim functional groups after cyclization is of some interest.

**Figure 11:**
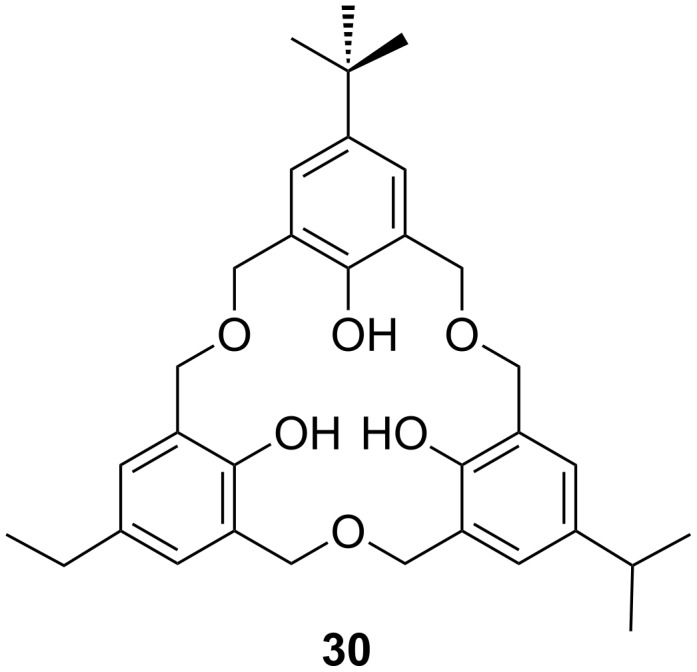
A chiral oxacalix[3]arene [45].

**3.2.1 Asymmetric oxacalix[3]arenes:** Using the synthetic routes described by Gutsche or Hampton it is possible to create oxacalixarenes with a range of upper-rim groups [14–15]; however, these methods can only yield threefold symmetric oxacalix[3]arenes. In order to introduce other groups and create asymmetric derivatives it is necessary to go through a stepwise synthetic route. Fortunately the strategy described by Fuji in 1998 [19] can be used to prepare linear trimers in which two or three different substituents are present. Using this method it was possible to prepare chiral oxacalix[3]arenes incorporating *t*-Bu, iPr, Et or H in the *para*-position of the phenolic moieties, as seen in example **30** in Figure 11 [45].

**Figure 12:**
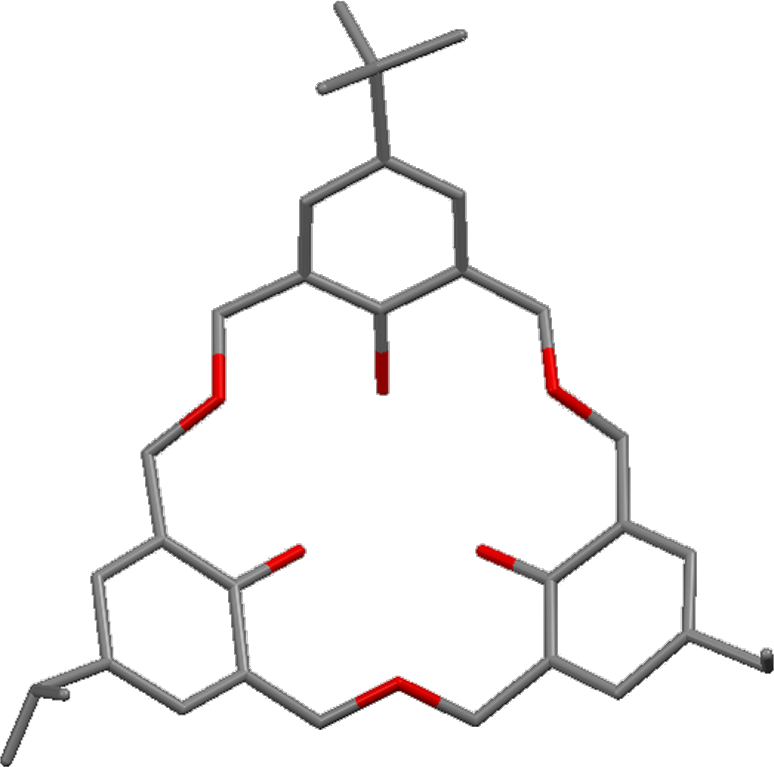
X-ray crystal structure of asymmetric oxacalix[3]arene **30** incorporating *t*-Bu, iPr and Et groups (CCDC ID 108839) [19].

The enantiomers can be separated by a chiral HPLC column and give opposite circular dichroic spectra, and can be crystallized out for structural characterization. X-ray crystallography was again able to determine the structure of compound **30** (Figure 12).

**Scheme 17:**
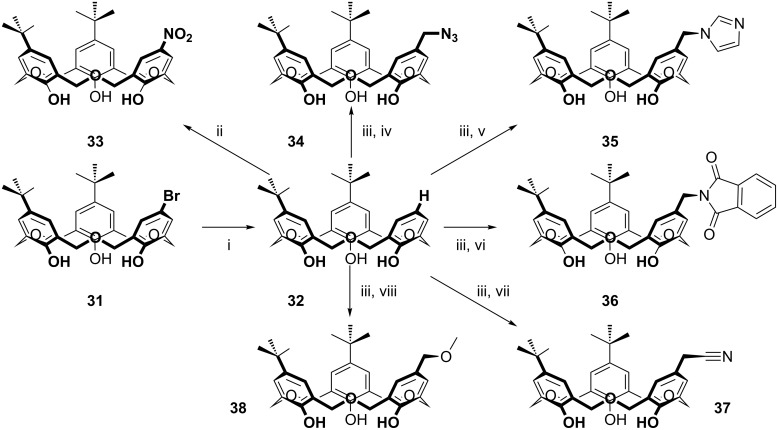
Reactions of an oxacalix[3]arene incorporating an upper-rim Br atom with (i) Pd(OAc)_2_, PPh_3_, HCO_2_H, Et_3_N; (ii) NH_4_NO_3_, acetic anhydride; (iii) Et_2_NH, H_2_CO (aq), AcOH, MeI; (iv) NaN_3_; (v) imidazole; (vi) potassium phthalimide; (vii) NaCN; (viii) NaOMe [46].

The work was extended in 2001 [21], and expanded in 2002 [46] to include a single Br substituent (**31**), which led to an important advance in oxacalix[3]arene chemistry as debromination of **31** allowed the introduction of new groups in the vacant *para*-position via the mono-unsubstituted derivative **32** as shown in Scheme 17. The route introduced nitro (**33**), azide (**34**), imidazole (**35**), phthalimide (**36**), cyano (**37**) and methoxyether (**38**) groups, linked to one of the oxacalix[3]arene rings by a methylene spacer.

As noted earlier, in Scheme 3, Komatsu’s diformylphenol approach also generates symmetric and asymmetric oxacalix[n]arenes, where *n* = 3 or 4 [22].

**Scheme 18:**
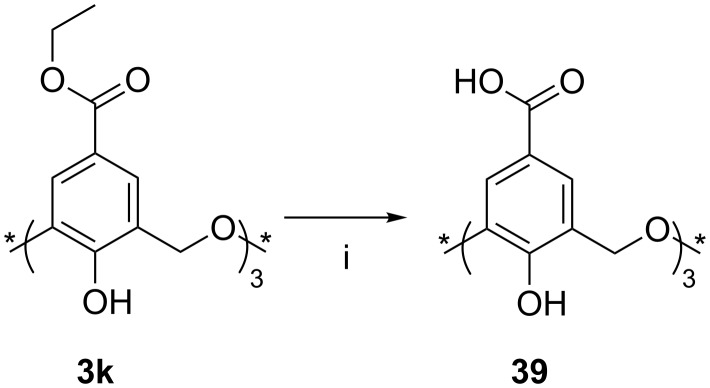
Synthesis of acid **39**: (i) NaOH, EtOH/H_2_O, HCl (aq) [47].

**3.2.2 Upper-rim esters and their reactions:** Formation of the oxacalix[3]arene **3k** with an upper-rim ester [47–49] makes further derivatives accessible by cleavage of the ester to leave the carboxylic acid **39** as shown in Scheme 18.

**Figure 13:**
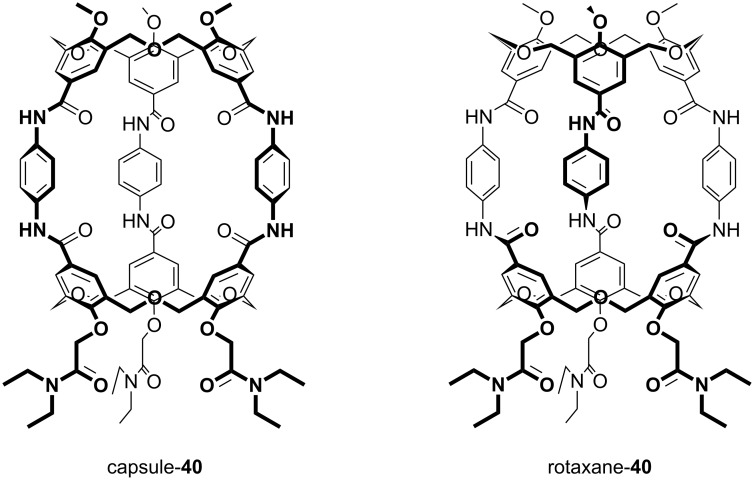
Two forms of dimeric oxacalix[3]arene **40** [47].

Shinkai used this methodology to prepare dimeric oxacalix[3]arene capsules linked by 1,4-xylylenediamine spacers. Derivatives of **39**, protected at the lower rim by methyl or *N*,*N*-diethylamide groups, were coupled to mono-*t*-Boc-protected 1,4-xylylenediamine. Subsequent deprotection and reaction with a second equivalent of the oxacalixarene acid gave the dimeric compound (capsule-**40**) shown in Figure 13. A nonencapsulating analogue was prepared through reaction of the acid derivative with benzylamine. The overall yield from the oxacalix[3]arene is less than 5%, but, given that the dimerization proceeds in only 14%, this is nevertheless quite impressive. However, in addition to the formation of the molecular capsule, a self-threaded dimer (rotaxane-**40**) was also isolated, which had resulted from an upper-rim substituent threading through the central cavity during dimerization. The existence of the rotaxane structure was deduced from the complexity of the patterns observed in the ^1^H NMR spectrum compared to that of the capsule. A similar strategy was adopted to incorporate porphyrin linkers between two oxacalix[3]arenes, but, due to the size of the porphyrins and their rigidity, only the capsular form was found [50]. Treatment with zinc(II) acetate introduced three equivalents of the metal, one for each porphyrin unit.

**Scheme 19:**
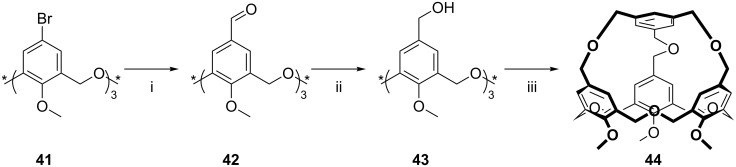
Capping the upper rim: (i) *t*-BuLi, THF, −78 °C; (ii) NaBH_4_, THF/EtOH; (iii) 1,3,5-tris(bromomethyl)benzene, Na_2_CO_3_, DMF [51].

**3.2.3 Capping the upper rim:** Capping the upper rim is also possible, as shown by Araki in 2000, through a complex synthetic pathway starting from bromooxacalix[3]arene [51]. As shown in Scheme 19, oxacalix[3]arene **3f** was treated with methyl iodide in the presence of NaH in THF at reflux to afford its methyl ether **41** in 41% yield. With the lower rim protected, the upper rim was converted to the aldehyde **42** and then reduced to the methylol **43**. Reaction with 1,3,5-tris(bromomethyl)benzene in a boiling suspension of NaH in THF/DMF afforded the upper-rim capped compound **44** in 26% yield. The sulfur-bridged analogue **45** was prepared in 36% yield by bromination of the methylol-terminated oxacalix[3]arene, employing PBr_3_, and coupling with 1,3,5-tris(methanethiol)benzene in the presence of Cs_2_CO_3_ in THF.

**Figure 14:**
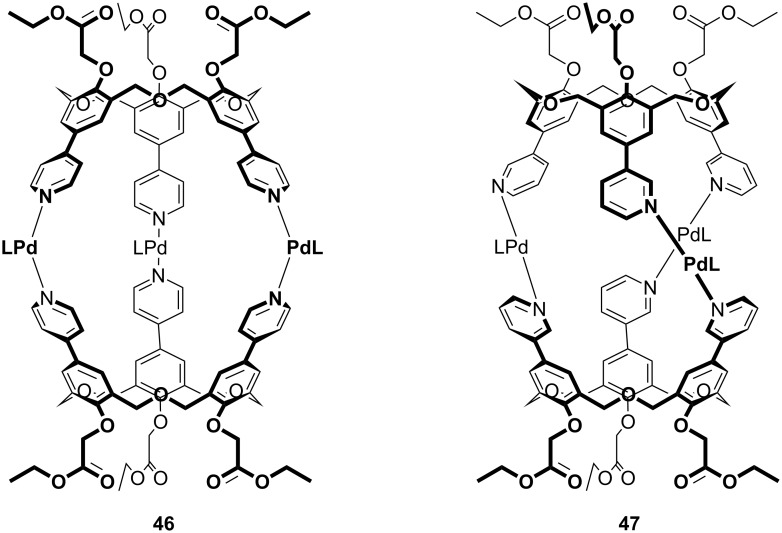
Oxacalix[3]arene capsules **46** and **47** formed through coordination chemistry [52–53].

**3.2.4 Upper-rim coordination chemistry:** The functionalization of the upper rims of oxacalix[3]arenes has also been achieved through classical inorganic coordination chemistry. Shinkai reported that the reactions of 4- and 3-pyridyloxacalix[3]arenes, protected on the lower rims by esters or methyl ethers, with [1,3-(diphenylphosphine)propane]palladium(II) salts gave dimeric capsules linked by three Pd(II) ions at the 4-pyridyl groups (**46**, Figure 14) or 3-pyridyl groups (**47**) [52–53]. The twist inherent in pyridylphenols, and by extension oxacalix[3]arenes incorporating these motifs, was expected to result in two chiral (*M* and *P*) forms of the capsules. The addition of Na^+^ appeared to enhance the twisting of capsule **46**, presumably through an allosteric effect that occurred when the cations bound to the lower-rim esters, as indicated by increasingly complex ^1^H NMR patterns. When **46** bound to *S*-2-methylbutylammonium triflate, the presence of a chiral complex was confirmed by circular dichroism [53].

### 4 Oxacalix[3]arene complexes

#### 4.1 Complexation by parent oxacalix[3]arenes

**4.1.1 Receptors for ammonium cations:** The symmetric cavity of the oxacalix[3]arenes, with three CH_2_OCH_2_ bridges and electron-rich aromatic groups, makes them attractive macrocycles to bind ammonium cations. The affinity of **3a** for acetylcholine and several other quaternary ammonium ions was investigated by Masci in 1995 [54] who found that *K*_assoc_ values in CDCl_3_ were modest, ranging from 38 M^−1^ for *N*,*N*,*N*-trimethylanilinium to 90 M^−1^ for *N*,*N*-dimethylpyrrolidinium, but significantly greater than those of the dihomocalix[4]arene and tetrahomooxacalix[4]arene analogues.

**4.1.2 Alkali-metal complexes:** The parent oxacalix[3]arenes (calixarenes with free OH groups) show little ability to bind alkali metals, and extraction studies from water to CH_2_Cl_2_ showed that this ability was enhanced only in the presence of strong bases [15]. Hampton’s purification of **3a** involved the formation and precipitation of the Na^+^ salt, which would seem to indicate a significant affinity for metal cations. Surprisingly, only *para*-chlorooxacalix[3]arene, **3e**, was found to bind alkali metals and then only when triethylamine was used to promote salt formation. The binding constants were determined by ^1^H NMR as 0.39 M^−1^ for Na^+^, 0.32 M^−1^ for K^+^ and 0.11 M^−1^ for Li^+^ in the presence of 10 equiv of the triflate salts. However, those oxacalixarenes form stronger complexes with transition, lanthanide and uranyl cations.

Cragg employed the quartz-crystal-microbalance technique to investigate binding by Na^+^, K^+^ and Ca^2+^ to **3a** and **3k** [48]. Again, Na^+^ was bound preferentially, with computer models suggesting that this was due to the depth to which the cation was drawn into the macrocyclic cavity when in the *cone* conformer.

**Figure 15:**
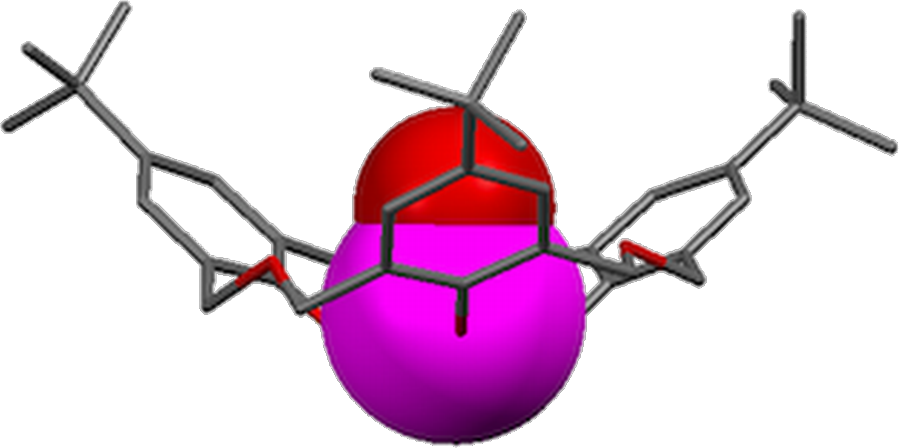
X-ray crystal structure of the **3b**-vanadyl complex (CCDC ID 240185) [57].

**4.1.3 Transition-metal complexes:** The first example of transition-metal binding to an oxacalix[3]arene was Hampton’s variable temperature ^1^H NMR investigation of the interactions between titanium(IV) species and **3a** [55]. In the absence of crystallographic evidence the NMR splitting patterns were compared to simulated spectra. At ambient temperature the NMR-derived symmetry was *C*_3_*_v_*, matching that of the macrocycle, but upon cooling an asymmetric *C_s_* symmetry emerged. It was proposed that rapid interconversion between isomers occurred by a “turnstile” or Berry-pseudorotation mechanism. A subsequent paper from the group reported the crystal structure of the titanium(IV) isopropoxide (Ti(iPrO)_4_) complex [56]. The structure was dimeric; each macrocycle was present as the trianion bound to the titanium by all three oxygens and pulled slightly into the cavity by iPrO^−^. The paper also reported the result of a reaction between the lithium salt of **3b** and vanadyl chloride (VOCl_3_). Based on powder diffraction and ^51^V NMR data it was proposed that the VO group bound within the macrocyclic cavity, by analogy to the Ti(IV) complex, and that these units formed linear aggregates held together by V=O**···**V=O interactions. Ten years later, Redshaw was able to prove Hampton’s assertion regarding the structure by X-ray analysis of the VO complex shown in Figure 15 [57].

**Scheme 20:**
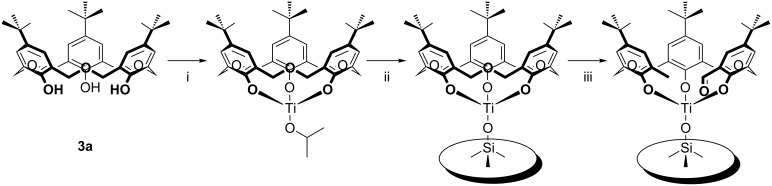
Effect of Ti(IV)/SiO_2_ on **3a**: (i) Ti(OiPr)_4_, toluene; (ii) triphenylsilanol, toluene; (iii) partially dehydroxylated silica gel, toluene [58].

Katz used calixarenes to disperse reactive titanium on silica in order to prepare a catalytically active surface [58]. While *p*-*tert*-butylcalix[4]arene appeared to work successfully, oxacalix[3]arene **3a** first bound titanium and was then cleaved to give an acyclic surface-bound product with free methyl and aldehyde termini (Scheme 20).

**Figure 16:**
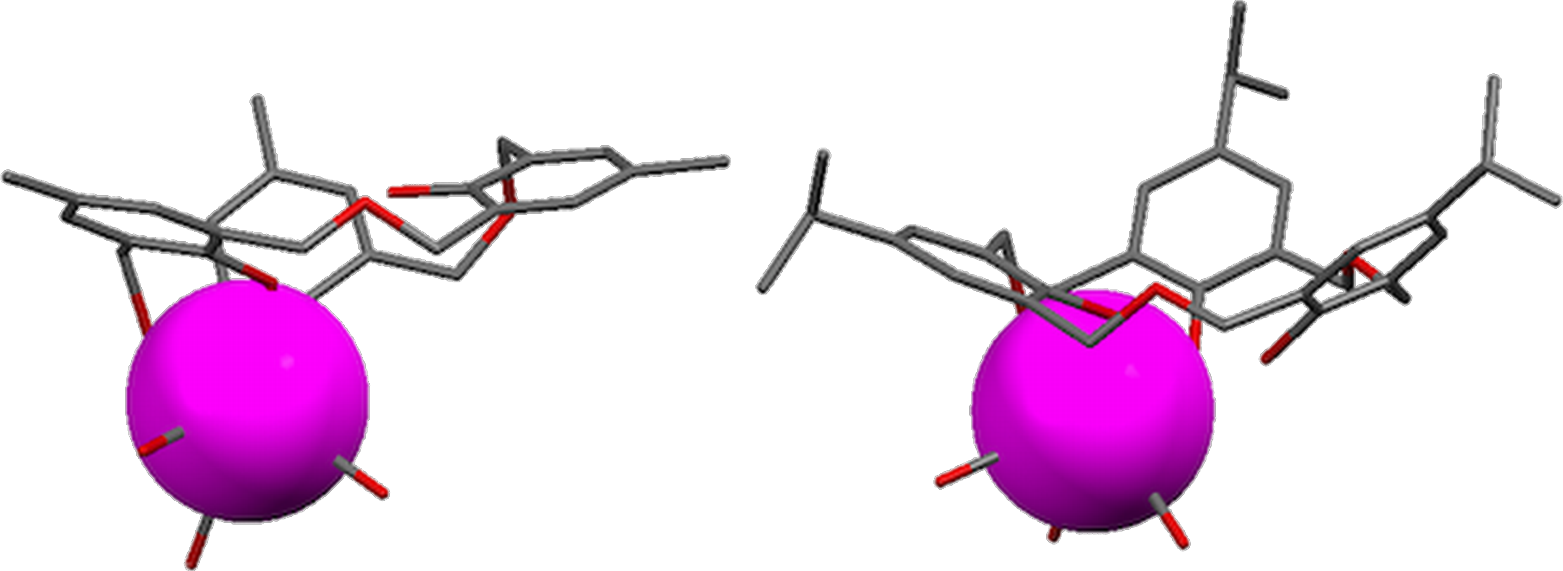
X-ray crystal structures of oxacalix[3]arene complexes with rhenium: **3b**∙Re(CO)_3_ (CCDC ID 620981, left) and **3d**∙Re(CO)_3_ (CCDC ID 620982, right) [49].

Klufers prepared complexes of **3b**, **3d** and **3k** through reaction of the macrocycles with (Et_3_N)_2_[Re(CO)_3_Br_3_] in acetonitrile [49]. The X-ray crystal structures of the complexes with **3b** and **3d** showed binding by Re(CO)_3_ to two deprotonated phenolic oxygen atoms as shown in Figure 16. Reaction with ester derivative **3k** at 85 °C resulted in decomposition of the macrocycle.

**Figure 17:**
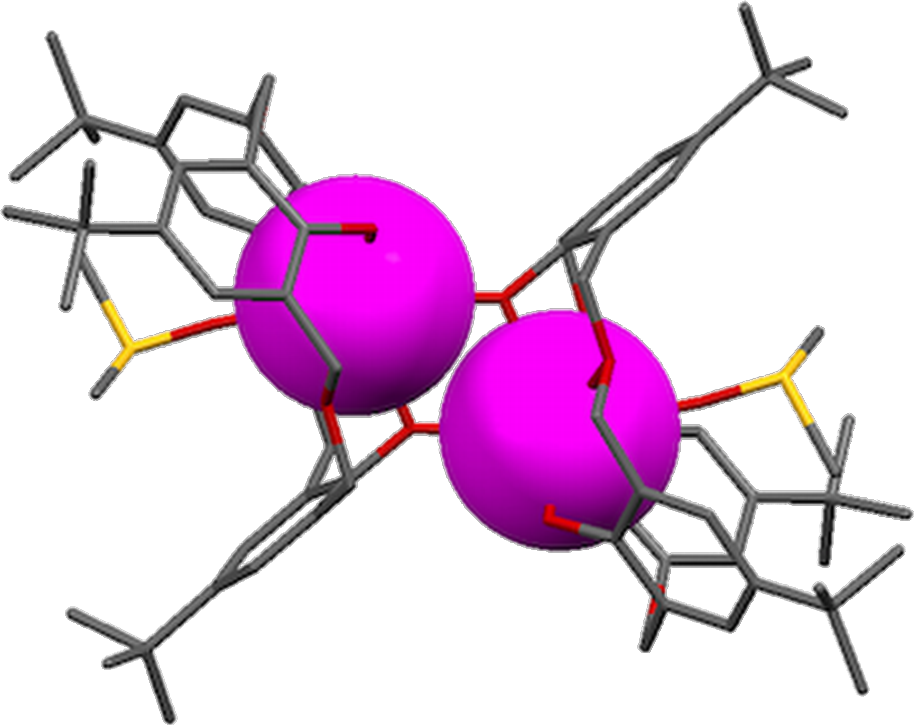
X-ray crystal structure of the La_2_·**3a**_2_ complex (CSD ID TIXXUT) [60].

**4.1.4 Lanthanide complexation:** The first study of the binding affinities of lanthanides for oxacalix[3]arenes was in 1995 when Hampton reported the crystal structure and dynamic behaviour of a scandium(III) complex of **3a** [59]. Later, the X-ray structures of lanthanum, lutetium and yttrium complexes with the same macrocycle showed 2:2 complexes between the cations and macrocycles [60]. In these structures the lanthanides are either six-coordinate, with distorted octahedral metal centres, or eight-coordinate, as in the structure illustrated in Figure 17.

The same group calculated the apparent binding constants of metal triflates with **3a** and **3e** [61]. Results showed that the binding constants for **3e** were slightly higher than **3a** and that the strength of binding increased in the sequence Ca^2+^, Na^+^, Li^+^ < Mg^2+^ < La^3+^ << Y^3+^ < Lu^3+^ << Sc^3+^. To reinforce this, the transporting ability of the oxacalixarenes was investigated. Aqueous/organic/aqueous liquid-membrane transport experiments were undertaken with both oxacalix[3]arenes in order to determine their cation selectivities. No transport of Li^+^ or Mg^2+^ was observed, but **3e** transported 44% of the Sc^3+^ over 24 h when a mixture of three cations (Sc^3+^, Mg^2+^ and Li^+^) was used as the source phase.

**Figure 18:**
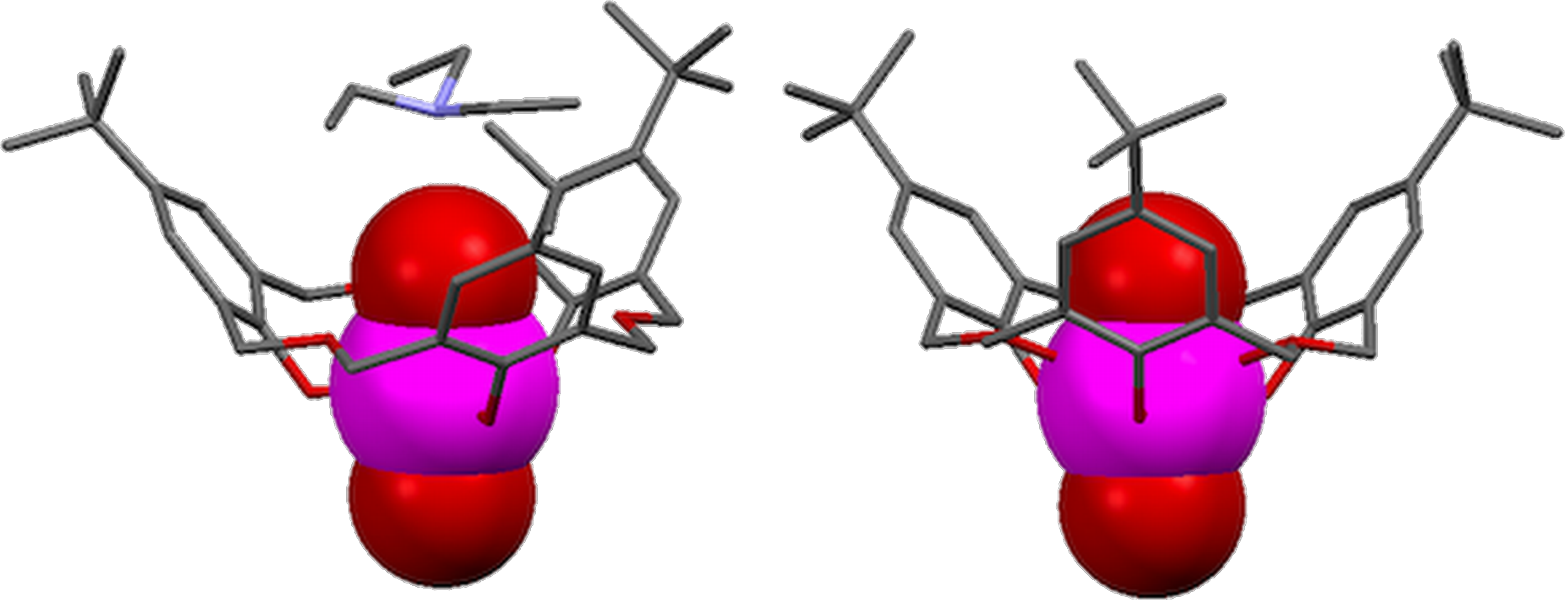
X-ray crystal structures of [**3a**∙UO_2_]^−^ with a cavity-bound cation (CCDC ID 135575, left) and without a coordinated cation (CCDC ID 181042, right) [62–63].

**4.1.5 Chelating behaviour with uranium:** Complexation of the uranyl cation by oxacalix[3]arenes has been ongoing since 1999 when Thuéry reported a complex of uranyl (UO_2_^2+^) and **3a** [62]. The X-ray crystal structure showed that the cation was threaded through a single macrocycle in what was, at the time, an unprecedented pseudotrigonal geometry, which included a weak interaction between the nitrogen of Et_3_N and a uranyl oxygen (Figure 18). Masci and Thuéry later reported more tetrahedrally and pentagonally distorted structures with **3a** and **3b** [63]. The nature of the alkylammonium counterion appeared to be influential in determining the final geometry around the uranium centre, yet in some cases it did not interact with the uranyl moiety (Figure 18).

**Figure 19:**
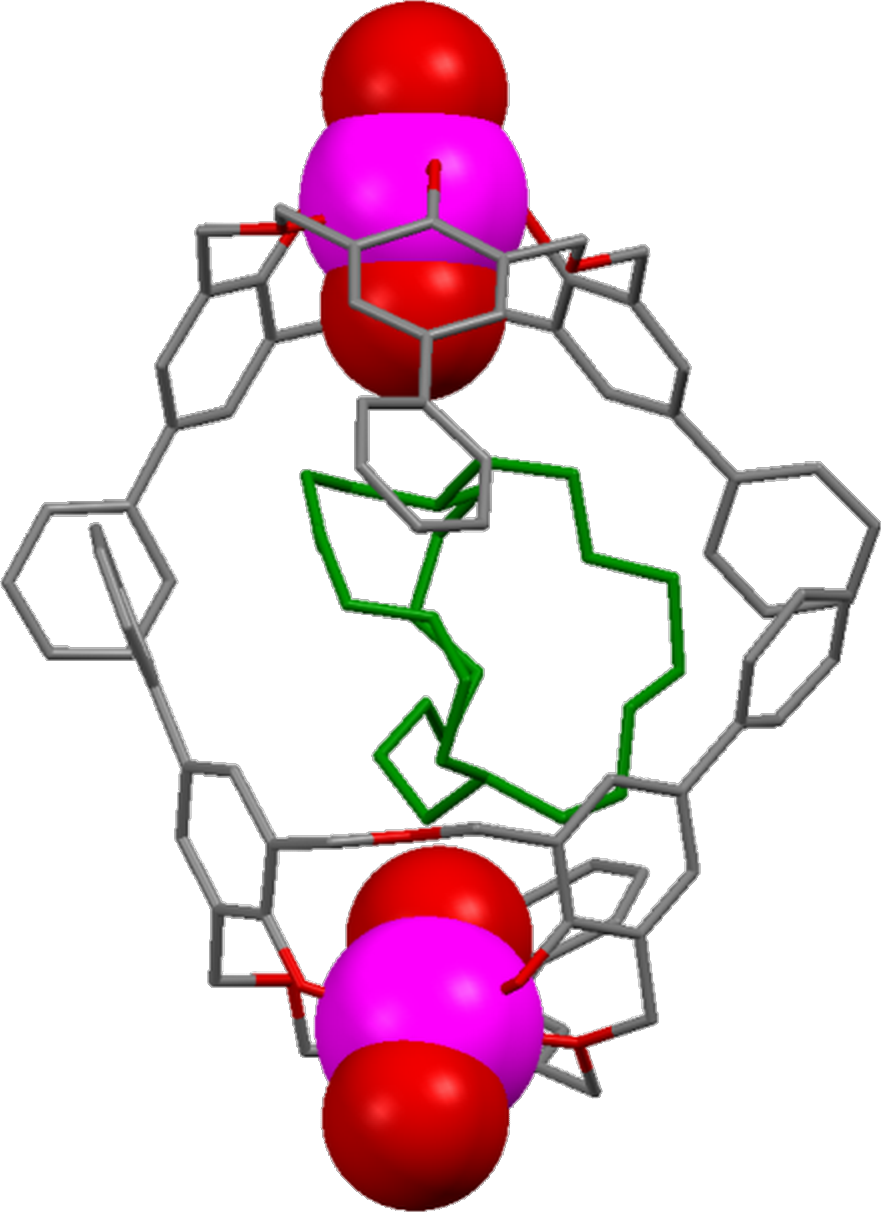
X-ray crystal structure of a supramolecule comprising two [**3g**·UO_2_]^−^ complexes that encapsulate a diprotonated cryptand (CCDC ID 181044) [64].

Replacing the alkylammonium cations with protonated [2.2.2]cryptand resulted in 1:1 and 2:1 complexes in which the uranyl–oxacalix[3]arene moiety acts as a recognition site for the [2.2.2]cryptand [64]. Figure 19 shows the crystal structure of the 2:1 complex.

#### 4.2 Binding properties of oxacalix[3]arene derivatives

One of the most important features of calixarenes in general and oxacalix[3]arenes in particular is their vast ability to selectively bind and carry ions and neutral species. This is achieved mainly with lower-rim derivatives in solution.

**4.2.1 Receptors for ammonium cations:** Although the simple parent oxacalix[3]arene **3a** is able to bind quaternary ammonium ions (as described above), several derivatives have also been studied with respect to these and other ammonium ions. Extraction studies from alkaline aqueous picrate solutions into CH_2_Cl_2_ indicated that the *n*-butyl ether derivative **10** showed a high affinity for *n*-BuNH_3_^+^ (82% *E*) as postulated by the authors, because both host and guest possess the same *C*_3_-symmetry [24]. Ethyl ester **12a** was more efficient at extracting *n*-BuNH_3_^+^ picrate from water into CH_2_Cl_2_ than its calix[4]arene analogue was, in both the *cone* (77% vs 24% *E*) and *partial-cone* (42% vs 6% *E*) conformers [29]. In a wider study, Yamato determined extraction data for **17a** with *n*-BuNH_3_^+^ picrate (98% *E cone* vs 93% *E partial-cone*), iBuNH_3_^+^ picrate (48% *E cone* vs 37% *E partial-cone*) and *t*-BuNH_3_^+^ picrate (35% *E cone* vs 14% *E partial-cone*) [34]. The hexaamide derivative **20** bound *n*-BuNH_3_^+^ well, and an anion dependence was determined; *K*_assoc_ values in CDCl_3_ were 536 ± 32 M^−1^ for Cl^−^ and 230 ± 17 M^−1^ for Br^−^ [37].

**Figure 20:**
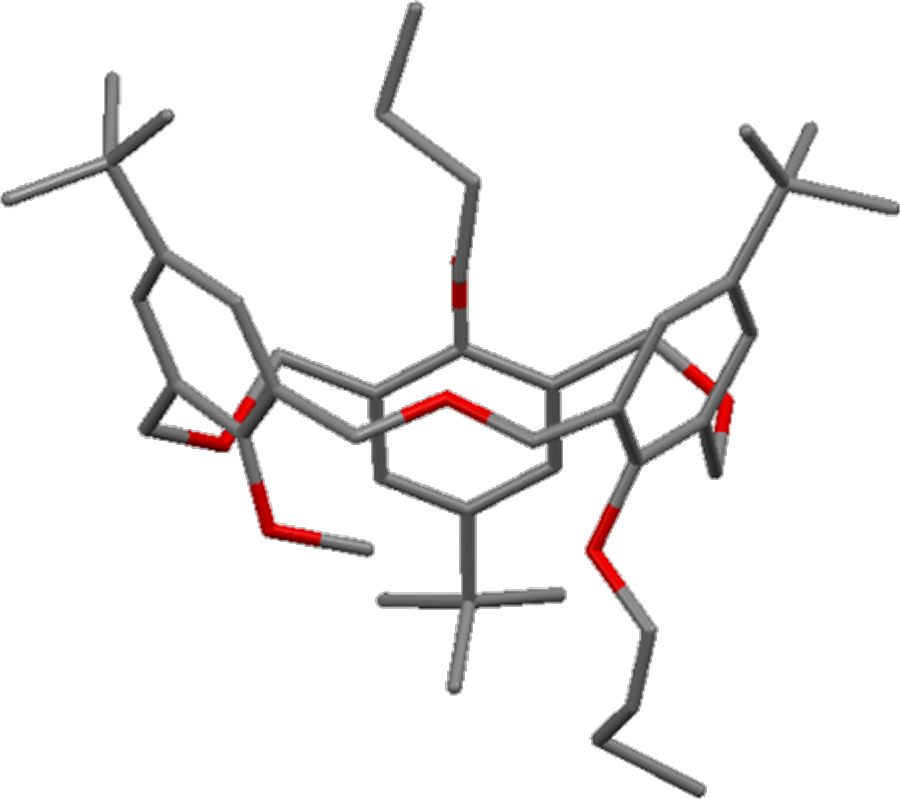
X-ray crystal structure of oxacalix[3]arene **49** capable of chiral selectivity (CSD ID HIGMUF) [65].

Studies of the *C*_3_ symmetrically capped triamide **13** reported that this derivative acts as a well-preorganized host for binding primary ammonium ions, such as phenylalanine methyl ester [31]. Chiral recognition of optically active primary alkyl ammonium ions was also obtained with an ether derivative of oxacalix[3]arene **3a** with one methyl and two *n*-butyl lower-rim substituents **49**, as shown in Figure 20 [65]. The compound was shown to exist in (+) and (−) enantiomers, and in a *partial-cone* conformation, proof of which came from X-ray crystallography. The compound bound to α-amino acid ethyl esters and 1-arylethylamines with the methoxy and one *n*-butoxy oxygen. The (−)-4-*tert*-butyloxacalix[3]arene derivative bound L-alanine ethyl ester and L-phenylalanine ethyl ester better than their enantiomers, with association constants of 4500 M^−1^ and 2000 M^−1^, respectively. (*R*)-1-Phenylethylamine and (*R*)-1-naphthylethylamine cations were bound more strongly by the (+)-enantiomer.

Allosteric effects can also be employed to affect the binding of ammonium cations. Katoh’s *N*-hydroxypyrazinone-containing oxacalix[3]arene **23** extracted *n*-BuNH_3_^+^ picrate and *t*-BuNH_3_^+^ picrate better in the presence of Ga^3+^, indicating cooperation between the two binding sites [38]. The association constant for *n*-HexNH_3_^+^ picrate was found to be 4375 M^−1^, but when Ga^3+^ was present this dropped to 2833 M^−1^, suggesting that the macrocyclic cavity, while preorganized for the smaller cations, was too rigid for the extended ammonium cation.

**Figure 21:**
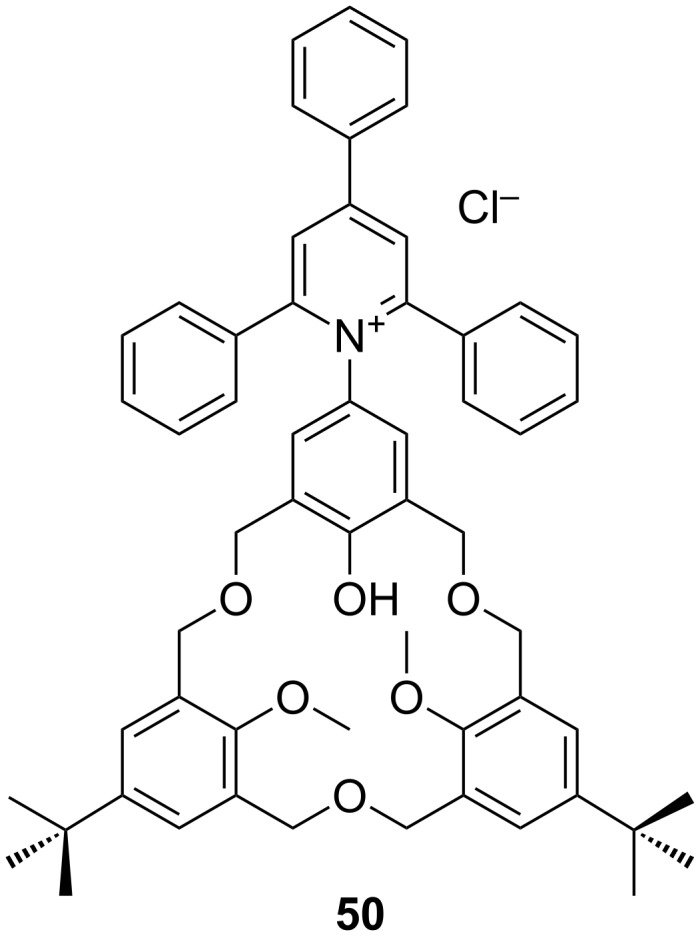
The structure of derivative **50** incorporating a Reichardt dye [66].

One of the more unusual derivatives to have been prepared, **50**, incorporates an *N*-pyridinium dye on one of the upper-rim positions, which, in combination with the phenolic unit of the macrocycle, forms a proton-ionizable Reichardt dye, illustrated in Figure 21 [66]. The other *p*-*tert*-butyl substituted phenols are blocked from ionization, as are the methyl ethers. The native oxacalix[3]arene dye is pale green and gives no response to benzylamine (BzNH_2_) or triethylamine (Et_3_N), but cyclohexylamine (*c*-HexNH_2_) and *n*-butylamine (*n*-BuNH_2_) bind with a concomitant colour change to blue.

**4.2.2 Alkali metals:** The ionophoric properties of the conformationally mobile ethyl ether of **3a** (**8**) and both *cone* and *partial-cone n*-butyl ether **10** derivatives toward alkali-metal cations were estimated by extraction experiments from alkaline aqueous picrate solutions to CH_2_Cl_2_ [24], with the latter showing some preference for K^+^ (59% *E*) over Na^+^ (6%) and Cs^+^ (35%).

Replacement of the alkyl groups by residues with additional binding sites, such as the carbonyl group, strongly affects the binding ability of calixarene derivatives. Thus, the binding properties of derivatives containing esters, amides and ketones, have been assessed. Extraction studies performed under the same conditions as described above reported that *cone* ester **12a** shows high selectivity for Na^+^ whereas the *partial-cone* conformer shows K^+^ selectivity (Table 1) [29]. Similar extraction experiments performed with amide **17a** [34] reported that this derivative is a better phase-transfer agent than **12a**, but shows the same trend as **12a**: *cone*-**17a** exhibits the highest preference for Na^+^, while *partial-cone*-**17a** prefers K^+^ (Table 1).

**Table 1:** Percentage extraction of alkali-metal picrates into CH_2_Cl_2_.^a,b^

	Li^+^	Na^+^	K^+^	Cs^+^

*cone*-**12a**	7	79	64	49
*paco*-**12a**^b^	0	26	88	82
*cone***-17a**	–	93	72	–
*paco*-**17a**	–	28	73	–

^a^Data adapted from references [29] and [34]. ^b^*partial-cone* denoted as *paco*.

The association constants, *K*_assoc_, for both derivatives (**12a** and **17a**) were determined in THF/CHCl_3_ (1:1) at 25 °C by UV absorption spectrophotometry (Table 2) [25].

**Table 2:** Association constants (log *K*_assoc_) of alkali- and alkaline-earth-metal complexes.^a,b^.

	Na^+^	K^+^	Rb^+^	Cs^+^	Mg^2+^	Ca^2+^	Ba^2+^

*cone*-**12a**	4	4.7	4.2	3.9	<2	<2	<2
*cone*-**17a**	>7	5.9	5.5	5.2	4.9	>7	>7
*paco*-**17a**^b^	5.1	6.2	6.0	5.5	–	–	–

^a^Data adapted from reference [25]. ^b^*partial-cone* denoted as *paco*.

Marcos [39,67] reported binding data for alkali- and alkaline-earth-metal cations with **17a** and **24** (Table 3). Extraction studies performed under different conditions than the previous ones (neutral aqueous picrate solutions to CH_2_Cl_2_), indicated that both derivatives show similar extraction profiles, although **17a** is a much stronger binder than **24**. Both exhibit highest selectivity for Na^+^ (50 and 20% *E* for **17a** and **24**, respectively) and **17a** is also a good extractant for Ba^2+^ (55% *E*).

**Table 3:** Percentage extraction of alkali and alkaline earth metal picrates into CH_2_Cl_2_.

	Li^+^	Na^+^	K^+^	Rb^+^	Cs^+^	Mg^2+^	Ca^2+^	Sr^2+^	Ba^2+^

*cone*-**17a**^a^	25	50	32	27	20	17	34	41	55
*cone*-**24**^b^	4	20	5	6	6	2	4	4	4

^a^Data adapted from references [67]. ^b^Data adapted from reference [39].

Derivatives with heteroatoms on the lower rim have also been tested as cation chelators. The binding properties of 2-pyridylmethyloxy derivative **11a** in both conformations, have been established [27,68]. Extraction studies from neutral aqueous picrate solutions to CH_2_Cl_2_ showed that, among all the cations studied, the *partial-cone* conformer is a better extractant than the *cone*.

As well as simple oxacalix[3]arenes and their derivatives, capped compounds have also been investigated. Association constants for several metal cations were determined for Yamato’s lower-rim-capped derivative **18** [33]. Values were found for Na^+^ (log *K*_assoc_ 5.3), K^+^ (log *K*_assoc_ 6.7) and Cs^+^ (log *K*_assoc_ 5.8). This contrasts with log *K*_assoc_ of 7.6 for *n*-BuNH_3_^+^ picrate. The extractability of metals from aqueous solution into CH_2_Cl_2_ by Araki’s upper-rim-capped derivatives was also determined [51]. The complementary cavity size and the rigid structure of the cage molecule **44** probably led to the high Cs^+^ selectivity (≈45% *E*) compared to negligible amounts of Na^+^, K^+^ or Rb^+^ (<5% *E*); however, the sulfur-linked compound **45** failed to extract any cations.

**Figure 22:**
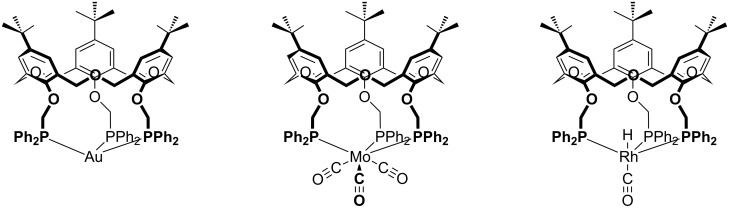
Phosphorylated oxacalix[3]arene complexes with transition metals: (Left to right) **26**∙Au, **26**∙Mo(CO)_3_ and **26**∙RhH(CO) [40].

**4.2.3 Transition metals:** The tris(diphenylphosphine) derivative **26** prepared by Matt [40] was reacted with [Mo(CO)_3_(cycloheptatriene)] to give a complex that was determined to be the symmetrically bound Mo(CO)_3_ complex involving all three of the phosphorus donors. The oxacalix[3]arene also formed a complex with rhodium. Elemental analysis supported a composition incorporating the H–Rh–C=O fragment. ^1^H NMR indicated that this was threaded through the macrocyclic annulus, based on the presence of a peak at −9.70 ppm, and infrared analysis showed a carbonyl absorption band at 1977 cm^−1^. This suggested an orientation in which the hydrogen was *endo*, and the carbonyl *exo*, to the macrocyclic cavity. Gold(I) and silver(I) complexes also form with the cations most likely adopting a trigonal planar *C*_3_*_v_* geometry, as shown in Figure 22, based on the symmetric ^31^P NMR pattern at ambient temperature. At lower temperatures, however, the A_3_X pattern seen for the silver(I) complex changes to an A_2_BX pattern, indicating that the apparent symmetry is a time-averaged effect.

**Figure 23:**
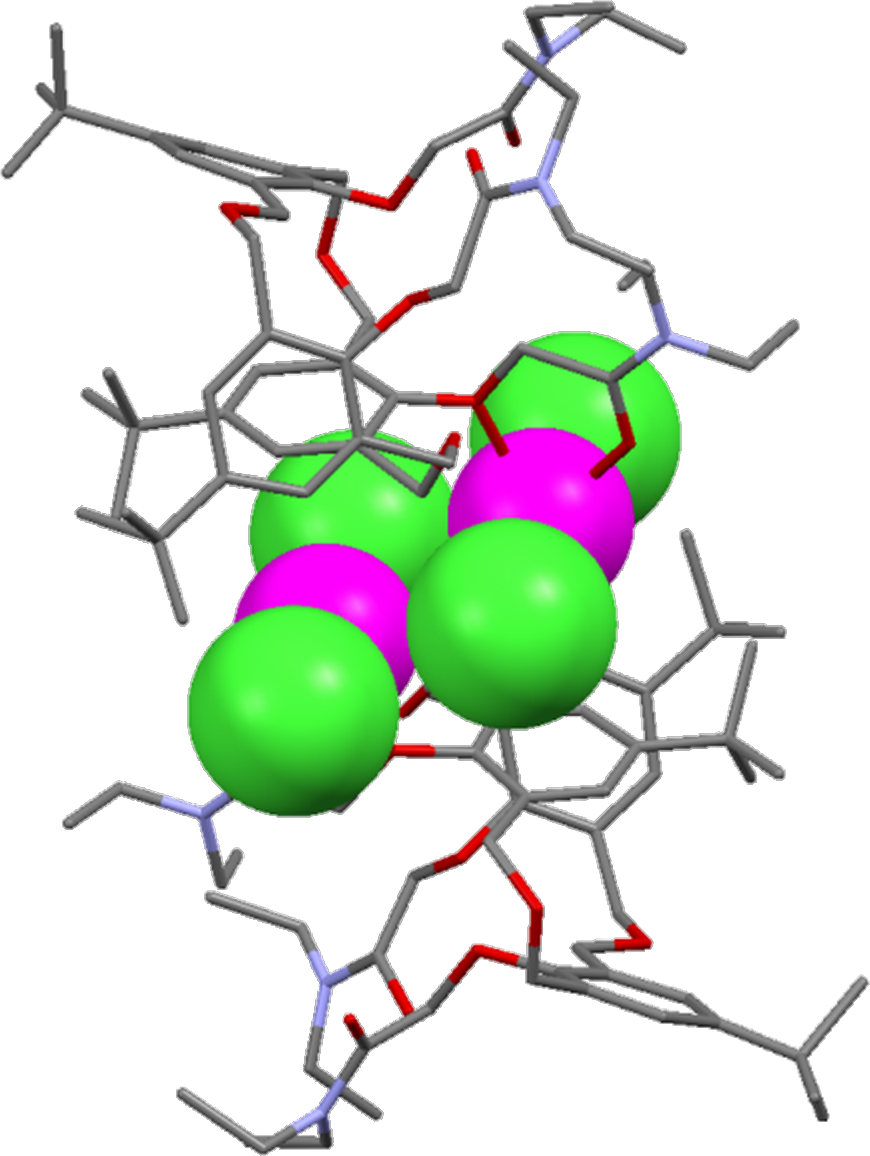
X-ray crystal structure of [**17a**·HgCl_2_]_2_ (CCDC ID 168653) [69].

Cragg reported the reaction of **17a** with mercury(II) chloride and the X-ray crystal structure of the product (Figure 23) [69]. The structure revealed that a [HgCl_2_]_2_ fragment bridged between two macrocycles through coordination to one amide group of each. The cations were thus *exo* to the macrocyclic cavity and represented the first example in which a cation was not bound within the annulus.

Marcos reported on the binding properties and theoretical studies of **17a** [67] and **24** [39] with transition and heavy metals. Extraction studies from neutral aqueous picrate solutions to CH_2_Cl_2_ indicated that amide **17a** is a good extractant for Ni^2+^, Co^2+^, and Ag^+^, and mainly for Pb^2+^ with 80% *E*. The data in Table 4 also shows that ketone **24** is a weak extracting agent, with a slight preference for Ag^+^. This is in agreement with the higher basicity of the carbonyl oxygen in the amide group compared with the ketone group.

**Table 4:** Percentage extraction of transition- and heavy-metal picrates into CH_2_Cl_2_.

	Mn^2+^	Fe^2+^	Co^2+^	Ni^2+^	Cu^2+^	Zn^2+^	Ag^+^	Cd^2+^	Pb^2+^

**17a**^a^	19	19	39	45	24	15	40	37	80
**24**^b^	2	5	2	4	4	3	13	3	5

^a^Data adapted from reference [67]. ^b^Data adapted from reference [39].

**4.2.4 Lanthanides:** Marcos investigated the lanthanide extraction by both **17a** [70] and **24** [39] using the same conditions as described above (Table 5). Ketone **24** is a poor phase-transfer agent (% *E* ranges from 5 to 7), while amide **17a** clearly discriminates between the light and heavy lanthanides. The lower-weight cations, such as Ce^3+^, Pr^3+^ and Nd^3+^ (34% *E*) are preferred over the heavier, such as Er^3^*^+^* and Yb^3+^ (13% *E*). The stability constants for the 1:1 complexes with **17a** were also determined by UV absorption spectrophotometry in methanol at 25 °C, by using chloride salts. The same positive discrimination for the light lanthanides was observed (log *β* = 5.5 and 3.4 for La^3+^ and Yb^3+^, respectively).

**Table 5:** Percentage extraction of lanthanide-metal picrates into CH_2_Cl_2_.

	La^3+^	Ce^3+^	Pr^3+^	Nd^3+^	Sm^3+^	Eu^3+^	Gd^3+^	Dy^3+^	Er^3+^	Yb^3+^

**17a**^a^	28	34	34	34	31	30	17	18	13	13
**24**^b^	6	5	5	6	6	6	6	5	6	7

^a^Data adapted from reference [70]. ^b^Data adapted from reference [39].

The complexing ability of the ionizable tricarboxylic acid **14a** towards lanthanides Pr^3+^, Eu^3+^ and Yb^3+^ and actinide Th^4+^ was established in methanol, by potentiometry measurements [71]. Results showed that the complex formed with Th^4+^ was more stable than the complexes of lanthanides (log *β* values are 20.5, 19.6, 21.3 and 23.1, respectively).

### 5 Other applications

#### 5.1 Hosts for fullerenes

One of the remarkable characteristics of calixarenes is the bowl shape of the molecule. In the case of oxacalix[3]arenes, the bowl is quite shallow, which indicates that they may be good hosts for spherical guests and immediately suggests binding to fullerenes. Furthermore, the macrocyclic bowl is the perfect size for C_60_ and has a complementary threefold-symmetry element.

Based on the knowledge that *p*-*tert*-butylcalix[8]arene was able to complex C_60_ [72–73] Shinkai investigated the interaction of C_60_ with **3a** in 1997 by UV–vis spectroscopy [74]. In a later full paper, UV–vis absorption spectra of C_60_ were recorded with calix[*n*]arenes and oxacalix[3]arenes. The interaction of fullerenes with calixarenes affected the spectra between 420 and 450 nm [75]. By using the Benesi–Hildebrand method, **3a** was shown to bind to C_60_ with a *K*_assoc_ of 35.5 M^−1^ in toluene at 25 °C; however, when methylated on the lower rim, no binding was observed. Molecular modelling was employed to illustrate how the shallow cavity of **3a** allowed for optimum interactions between the oxacalix[3]arene aromatic rings and C_60_.

While subtle spectroscopic features and computer models appeared to indicate fullerene binding, structural evidence was to be more compelling. In 1998, Fuji reported the solid-state structure of **3f** with C_60_ as proof of 1:1 binding [76]. Alignment of the oxacalix[3]arene *C*_3_ axis with the same symmetry axis of the fullerenes is observed. This arrangement maximizes the number of points of contact within the supramolecular complex, thereby enhancing the van der Waals interactions. In the same paper, the association constants of several oxacalix[3]arenes were calculated by the Rose–Drago method based on absorption features at 425 or 430 nm in toluene. The strongest binding was observed for **3a** (35.6 M^−1^) and the weakest for **3h** (9.1 M^−1^).

Although spectroscopic methods are widely used to determine host–guest association constants, Georghiou has argued persuasively that spectral changes in solution may be due to a combination of several factors, of which host–C_60_ complex formation is only one [77]. Consequently, reported *K*_assoc_ values determined by this method should be treated with some caution.

**Figure 24:**
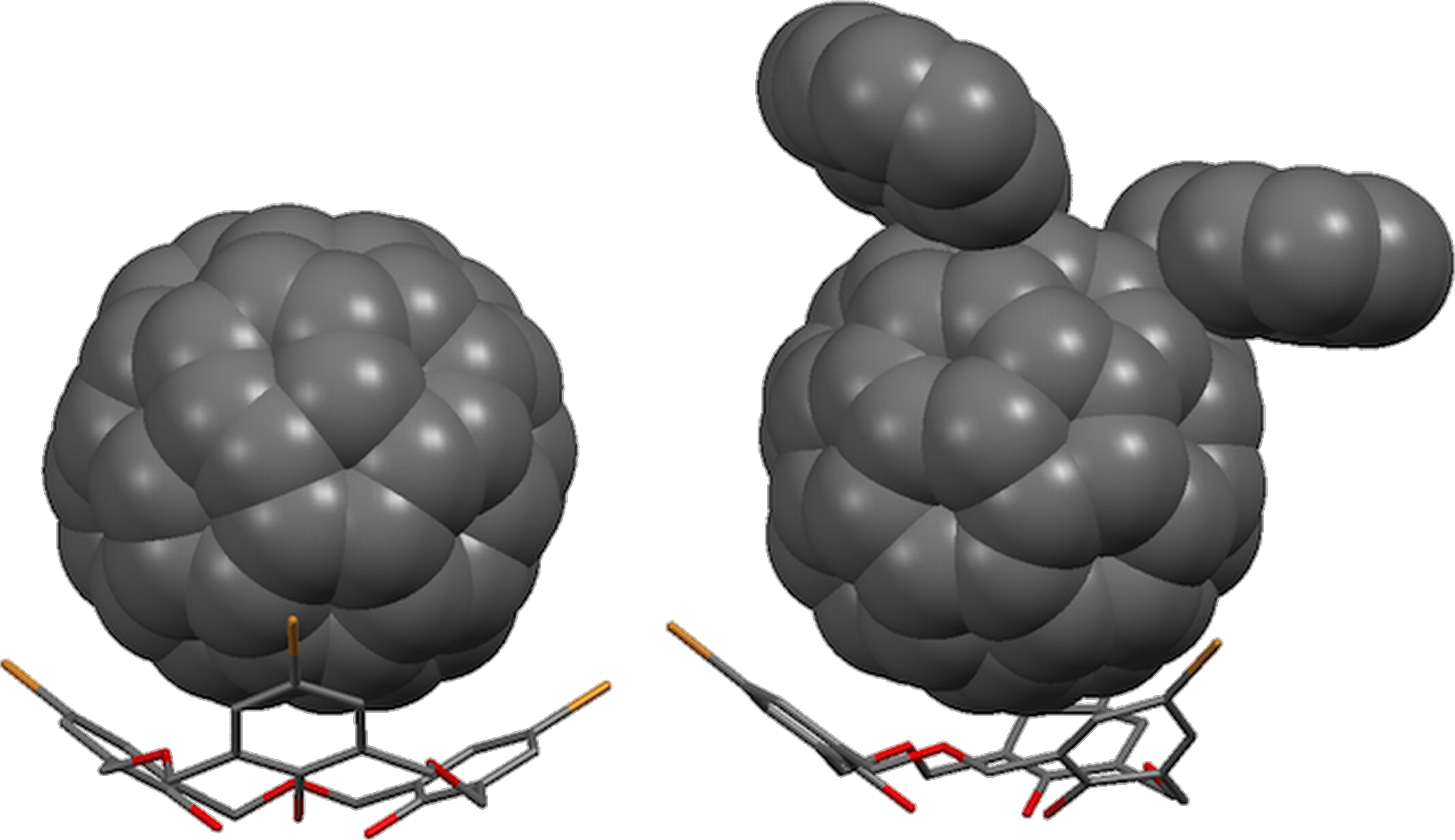
X-ray crystal structures of **3f** with C_60_ (CCDC ID 182801, left) [76] and a 1,4-bis(9-fluorenyl) C_60_ derivative (CCDC ID 139793, right) [78].

Fullerene derivatives that lack some of the symmetry of the parent compound have been shown to bind to oxacalix[3]arenes, as in Fuji’s X-ray structure of 1,4-bis(9-fluorenyl)-1,4-dihydro[60]fullerene with **3f** shown in Figure 24, in which the oxacalix[3]arene binds to the C_60_ derivative with the fluorenyl substituents oriented away from the macrocycle [78].

**Figure 25:**
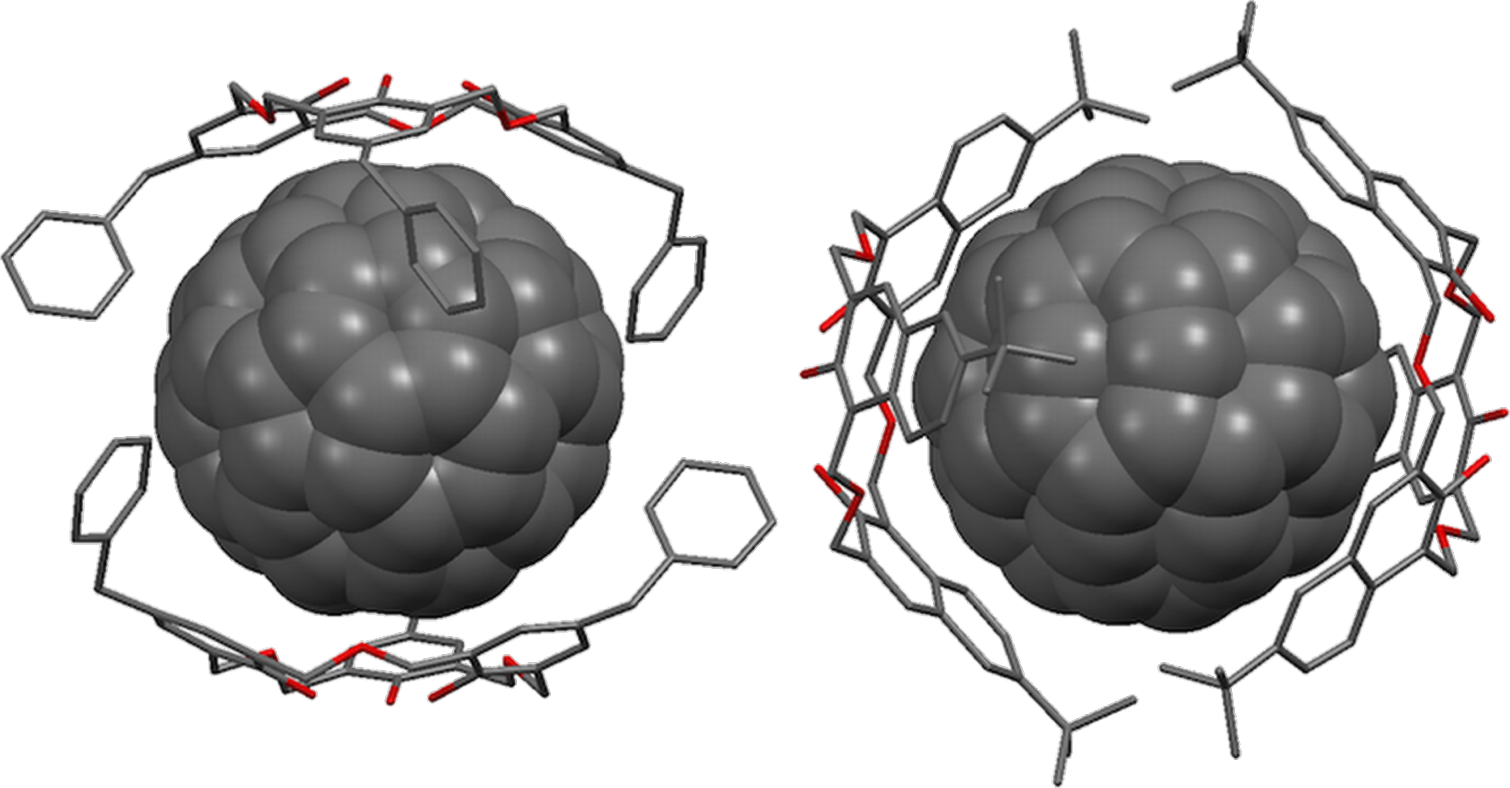
X-Ray crystal structure of **3i** and **6a** encapsulating C_60_ (CCDC ID 102473 and 166077) [23,79].

Raston reported that *p*-benzyloxacalix[3]arene (**3i**) formed a 2:1 complex with C_60_ in toluene [79]. The X-ray crystal structure showed how the two oxacalix[3]arenes bound on opposite sides of the fullerene, with their benzyl arms interdigitated. When the complex was isolated and added to CH_2_Cl_2_ then the fullerene was released. The method could be used to separate C_60_ from fullerite (a mixture of fullerenes of different sizes) in greater than 99.5% purity. A similar experiment was undertaken by Georghiou with **6a** leading to much higher association constants of 296 M^−1^ (toluene) and 441 M^−1^ (benzene) [23]. Crystallography revealed a similar interdigitated 2:1 complex to that observed by Raston for **3i** (Figure 25).

One area of interest has been the selective separation of C_70_ from a mixture of fullerenes. Komatsu proposed a method for the preferential precipitation of C_70_ over C_60_ with *p*-halooxacalix[3]arenes [80]. *p*-Iodooxacalix[3]arene (**3j**) was able to achieve 90% extraction with a selectivity approaching 90%.

**Figure 26:**
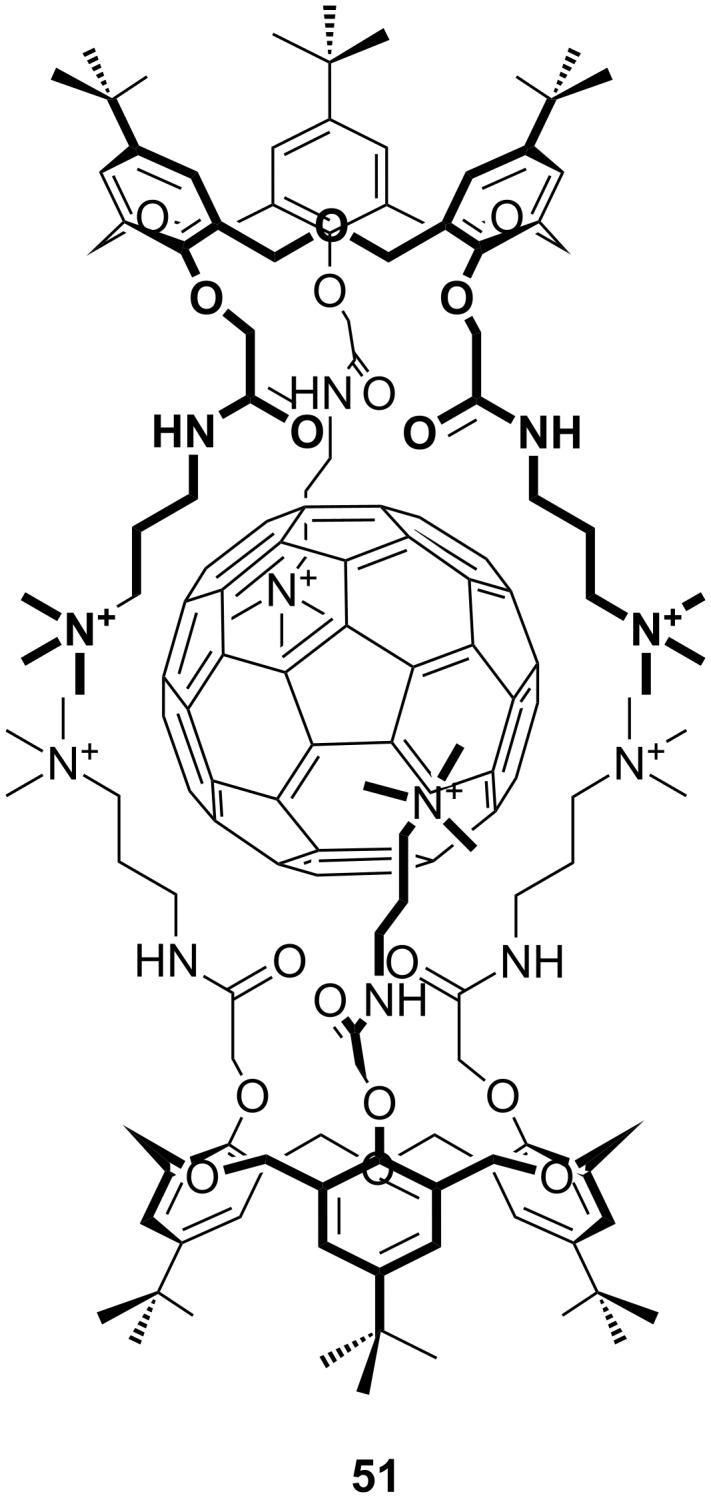
A C_60_ complexing cationic oxacalix[3]arene **51** [81].

An unexpected effect of fullerene complexation was that a water-soluble capsule formed from two *p*-*tert*-butyloxacalix[3]arenes with trimethylammonium groups on the lower rims, **51**, which bound C_60_, was able to cleave DNA (Figure 26) [81]. The capsule was solubilized as the MsO^−^ salt and applied to a supercoiled form of DNA. In the absence of light, no change was seen, but in the presence of visible light the DNA became “nicked”, that is, a phosphodiester bond in one strand was broken. The authors speculated that the cationic complex was able to bind to the anionic DNA whereupon ^1^O_2_ generated by photoinduced electron transfer from guanine and C_60_, or alternatively photochemically by C_60_ alone, cleaves the DNA strand. Ikeda later advanced this line of research to carbohydrate-containing oxacalix[3]arenes that functioned in water [82].

The same cationic complex was deposited as a monolayer onto an alkylsulfonate coated gold surface and elicited both a redox response, as determined by cyclic voltammetry, and a photochemical response to visible light [83–84]. The optical response was studied further [85], and a transient band was observed at 545 nm, which was not present in the spectrum of C_60_ alone. The origin of the band was ascribed to C_60_-capsule triplet–triplet absorption.

As discussed above, oxacalix[3]arenes with pyridine in the *para*-position and ethyl esters on the lower rim are able to form capsules through coordination to palladium [53]. Capsule **46** was shown to bind to C_60_ by the presence of two peaks in the ^13^C NMR spectrum, which did not coalesce even at 90 °C. ^1^H NMR was used to determine an association constant of 54 M^−1^ in Cl_2_CDCDCl_2_ at 60 °C. An asymmetric capsule incorporating an oxacalix[3]arene and three Zn(II)porphyrin moieties, **52**, was also able to bind C_60_ in a similar fashion with an association constant of 60 M^−1^ in toluene-*d*_8_ at −30 °C [86].

**Figure 27:**
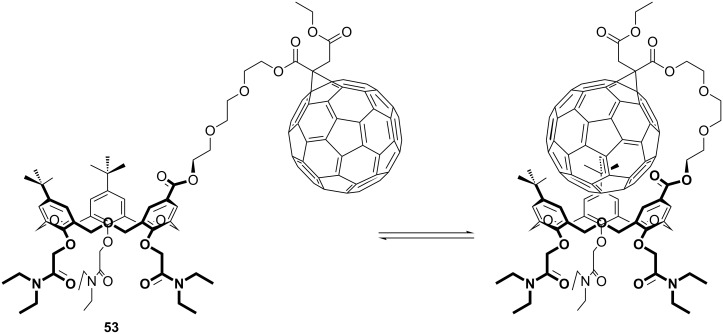
An oxacalix[3]arene-C_60_ self-associating system **53** [87].

Another strategy to promote fullerene inclusion in an oxacalix[3]arene was to link the two by a triethylene glycol tether to form a molecular cup-and-ball **53** [87]. In addition to self-inclusion, the authors also proposed the formation of higher order oligomers arising from C_60_ inclusion in a neighbouring oxacalixarene through the change in conformation illustrated in Figure 27.

#### 5.2 Fluorescent chemosensors

**Scheme 21:**
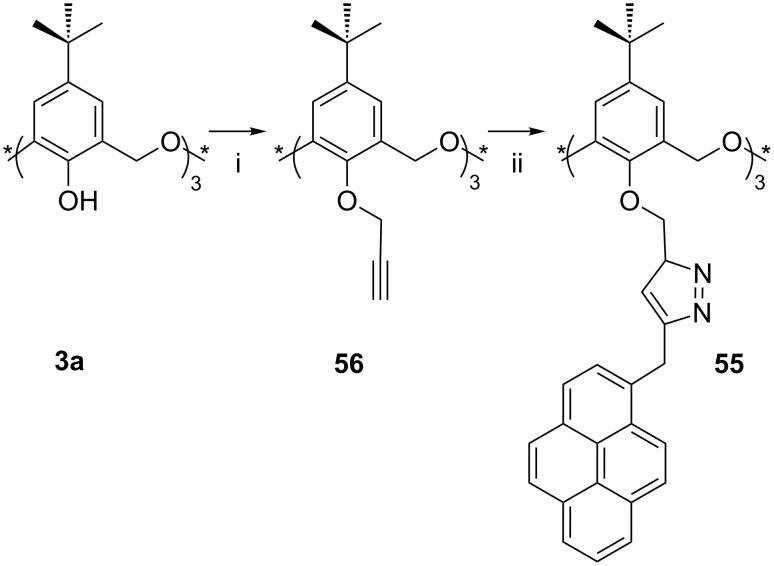
Synthesis of fluorescent pyrene derivative **55**: (i) Propargyl bromide, acetone; (ii) CuI, 1-azidomethylpyrene, THF/H_2_O [89].

In order to determine the equilibrium constants with quaternary ammonium ions, Shinkai [88] prepared an oxacalixarene with pendent pyrene groups, **54**, which fluoresced at 480 nm. Oxacalix[3]arene fluorescence was significantly quenched in the presence of *n*-hexyl ammonium cations (*n*-HexNH_3_^+^), but only in the *partial-cone* conformation, as the ammonium cation forced the lower-rim pyrene groups apart. The same cation had a much higher affinity for *cone*-**54** through its complementary binding sites, but approached these from the upper rim, leaving the excimer fluorescence unaffected. Yamato also pursued this path, preparing a tris(pyrenyl) derivative **55** in the *cone* conformer by employing “click” chemistry (Scheme 21) [89]. One interesting aspect of the synthesis was that the tris(propargyl) click precursor crystallized as a mixture of *cone* and *partial-cone* conformers, yet addition of *n*-BuNH_3_^+^ClO_4_^−^ to the conformers in solution pushed the equilibrium towards the *cone*. *Cone*-**55** gave a response to Pb^2+^ through the enhancement of minor fluorescence peaks between 370 and 400 nm, which were unaffected by other metal guests. The group also reported that the fluorescence intensity at 396 nm increased linearly when Zn^2+^ was added and that the 1:1 complex of this macrocycle gave an increasing linear response at 485 nm to H_2_PO_4_^−^ [90].

**Scheme 22:**
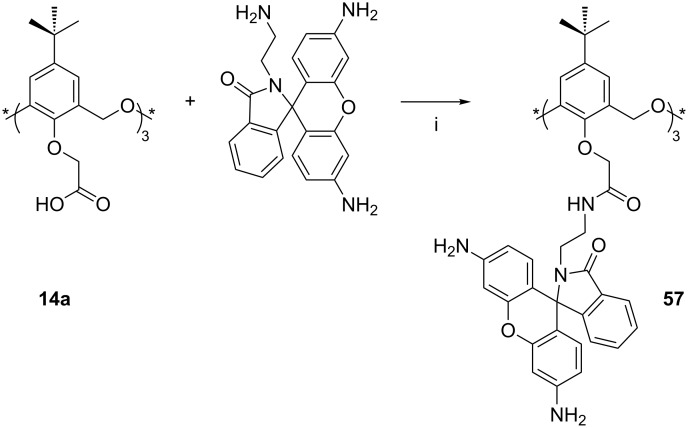
Synthesis of responsive rhodamine derivative **57**: (i) DCC, CH_2_Cl_2_ [91].

Rhodamine substituents can be introduced to the lower rim of the *cone*-**14a** through the ethylamine derivative of the dye (Scheme 22) [91]. Fluorescence enhancement was observed between 500 nm and 600 nm upon addition of Fe^3+^, Ni^2+^ and Sb^3+^ to **57**, turning the colourless solution fluorescent orange–yellow, together with a colourless-to-magenta colourimetric response.

**Scheme 23:**
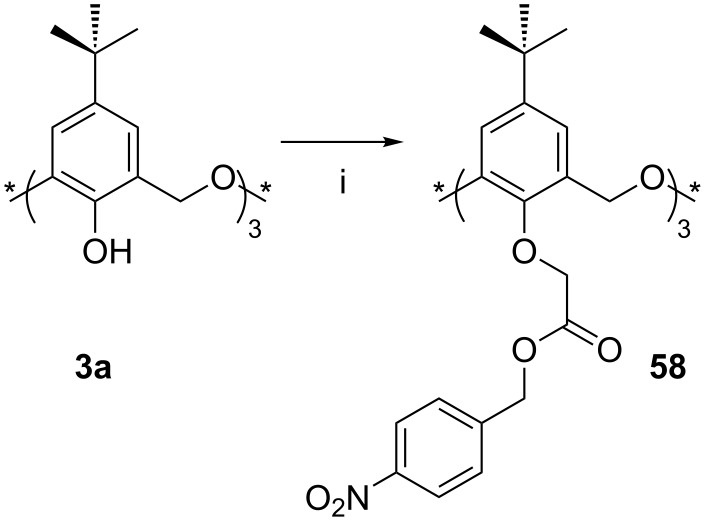
Synthesis of nitrobenzyl derivative **58**: (i) 1-Bromo-4-nitrobenzyl acetate, K_2_CO_3_, refluxing acetone, 3 h [92].

Kang found that the reaction of **3a** with 1-bromo-4-nitrobenzyl acetate gave the trisubstituted nitrobenzene derivative **58** in 40% yield (Scheme 23) as the *partial-cone* conformer [92]. When a range of fluorescent ammonium cations incorporating pyrene, anthracene or naphthalene groups was tested, quenching was observed. Association constants were determined to be in the range of 1850 M^−1^ to 78000 M^−1^. The uncharged pyrenemethylamine was not bound at all, and a trimethylpyrenium cation was weakly bound (*K*_assoc_ = 300 M^−1^).

#### 5.3 Ion-selective electrodes

Given the apparent oxacalix[3]arene selectivity for Na^+^ and certain protonated amines it is likely that they can act as ion-selective agents in electrodes. This aspect of oxacalix[3]arene research demonstrates that they are not limited to fluorescent sensor applications but can also function in the electrochemical sphere.

**5.3.1 Dopamine recognition:** The first example of oxacalixarenes being used as electrode modifiers was in 1999 when Odashima incorporated *cone p*-*tert*-butyloxacalix[3]arene tri(*n*-butyl ether) (**10**) in a PVC matrix liquid membrane [93]. The electrode displayed excellent selectivity for dopamine over biologically important alkali-metal cations K^+^, by a factor of 150, and Na^+^, by a factor of 1600. Selectivity for dopamine against other catecholamine neurotransmitters, such as adrenaline and noradrenaline, was also greater by a factor of at least 100. This selectivity obtained with **10** is a very promising result with the potential to be developed into a dopamine sensor for use under physiological conditions. The dopamine selectivity of the trimethyl ether analogue **7** was investigated by Arrigan at the interface between water and 1,2-dichloroethane using cyclic voltammetry [94]. The log *K*_assoc_ value obtained was 8.3, which was significantly higher than those for Na^+^ and K^+^. The log *K*_assoc_ comparative data for dibenzo-18-crown-6 were 7.6 for the dopamine complex and 10.1 for the K^+^ complex, indicating that not only was **7** a better host for dopamine but also that K^+^ would not be bound preferentially as is the case for the crown ether.

**5.3.2 Sensing Pb^2+^:** In 2007, Yaftian incorporated Matt’s phosphorylated derivative **26** in a membrane solution, prepared by dissolving PVC, NaBF_4_, a plasticizer and the oxacalixarene in THF, which was then used to coat a graphite electrode [95]. This electrode gave a good Nernstian response of 29.7 mV/decade, over a concentration range of 1 × 10^−8^ M to 1 × 10^−4^ M of Pb^2+^ ions, with a detection limit of 0.4 × 10^−8^ M. When tested in mixtures of several competing cations (such as alkali, alkaline earth, transition, heavy metal, lanthanide and Th^4+^ ions) the electrode was able to determine the concentration of Pb^2+^ correctly within 5%, even when other ions were present in tenfold excess.

Diethylacetamide **17a** was also used as an active material in ion-selective electrodes to check the detection of different types of cations [96]. Optimization of the PVC membrane composition was achieved by using different plasticizers (DEHA, *o*-NPOE and BBPA). The performance of the ISE incorporating **17a** indicated a high affinity for Pb^2+^ and the use of DEHA as the best plasticizer.

#### 5.4 Biological models

**Figure 28:**
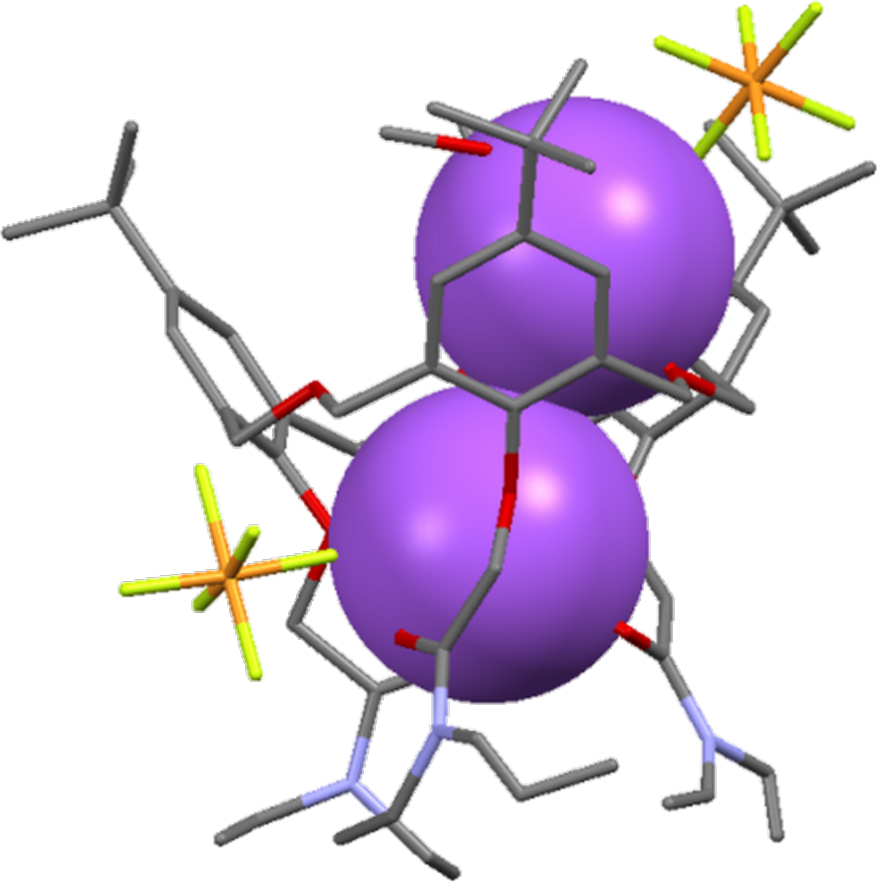
X-ray crystal structure of [Na_2_∙**17a**](PF_6_)_2_ (CCDC ID 116656) [97].

The crystal structure of the complex of **17a** with NaPF_6_ (Figure 28) shows how the lower-rim binding site, composed of phenolic oxygen and amide nitrogen atoms, is predisposed to bind Na^+^ in its ideal octahedral environment [97]. The compound has been proposed to be an artificial analogue for the filter region in cation channels formed by naturally occurring transmembrane proteins and has been shown to have some activity on transmembrane ion transport in cells.

## Conclusion

Since their origins in the phenol-formaldehyde chemistry of the 1960s, oxacalix[3]arenes and their analogues have shown themselves to be interesting and useful additions to the large array of artificial macrocycles that has been developed by supramolecular chemists. The *C*_3_ symmetry of oxacalix[3]arenes, commonly encountered in nature but relatively rare in synthetic host molecules, has made them valuable members of the calixarene family, with an affinity for guests with complementary binding requirements. While the parent compounds do not form particularly strong complexes with metal ions, their *O*-alkylated derivatives are easy to prepare and can show very efficient and selective cation binding, extending to alkyl ammonium salts. Advances in upper-rim functionalization allow for the formation of molecular capsules and chiral recognition sites, and applications have been found in fluorescence sensors, ion-selective electrodes and the extraction of pure C_60_ and C_70_ from crude fullerite. Fifty years on from their discovery by Hultzsch, oxacalix[3]arenes and their derivatives are still able to amaze chemists with their elegant symmetry and fascinating complexes.
